# Genome reduction and horizontal gene transfer in the evolution of Endomicrobia—rise and fall of an intracellular symbiosis with termite gut flagellates

**DOI:** 10.1128/mbio.00826-24

**Published:** 2024-05-14

**Authors:** Undine S. Mies, Vincent Hervé, Tom Kropp, Katja Platt, David Sillam-Dussès, Jan Šobotník, Andreas Brune

**Affiliations:** 1 Research Group Insect Gut Microbiology and Symbiosis, Max Planck Institute for Terrestrial Microbiology, Marburg, Germany; 2 Laboratory of Experimental and Comparative Ethology LEEC, UR 4443, University Sorbonne Paris Nord, Villetaneuse, France; 3 Faculty of Tropical AgriSciences, Czech University of Life Sciences, Prague, Czechia; 4 Biology Centre, Czech Academy of Sciences, Institute of Entomology, České Budějovice, Czechia; University of Hawaii at Manoa, Honolulu, Hawaii, USA

**Keywords:** termites, *Endomicrobiaceae*, Parabasalia, endosymbionts, lateral gene transfer, convergent evolution

## Abstract

Bacterial endosymbionts of eukaryotic hosts typically experience massive genome reduction, but the underlying evolutionary processes are often obscured by the lack of free-living relatives. Endomicrobia, a family-level lineage of host-associated bacteria in the phylum *Elusimicrobiota* that comprises both free-living representatives and endosymbionts of termite gut flagellates, are an excellent model to study evolution of intracellular symbionts. We reconstructed 67 metagenome-assembled genomes (MAGs) of *Endomicrobiaceae* among more than 1,700 MAGs from the gut microbiota of a wide range of termites. Phylogenomic analysis confirmed a sister position of representatives from termites and ruminants, and allowed to propose eight new genera in the radiation of *Endomicrobiaceae*. Comparative genome analysis documented progressive genome erosion in the new genus *Endomicrobiellum*, which comprises all flagellate endosymbionts characterized to date. Massive gene losses were accompanied by the acquisition of new functions by horizontal gene transfer, which led to a shift from a glucose-based energy metabolism to one based on sugar phosphates. The breakdown of glycolysis and many anabolic pathways for amino acids and cofactors in several subgroups was compensated by the independent acquisition of new uptake systems, including an ATP/ADP antiporter, from other gut microbiota. The putative donors are mostly flagellate endosymbionts from other bacterial phyla, including several, hitherto unknown lineages of uncultured *Alphaproteobacteria*, documenting the importance of horizontal gene transfer in the convergent evolution of these intracellular symbioses. The loss of almost all biosynthetic capacities in some lineages of *Endomicrobiellum* suggests that their originally mutualistic relationship with flagellates is on its decline.

**IMPORTANCE:**

Unicellular eukaryotes are frequently colonized by bacterial and archaeal symbionts. A prominent example are the cellulolytic gut flagellates of termites, which harbor diverse but host-specific bacterial symbionts that occur exclusively in termite guts. One of these lineages, the so-called Endomicrobia, comprises both free-living and endosymbiotic representatives, which offers the unique opportunity to study the evolutionary processes underpinning the transition from a free-living to an intracellular lifestyle. Our results revealed a progressive gene loss in energy metabolism and biosynthetic pathways, compensated by the acquisition of new functions via horizontal gene transfer from other gut bacteria, and suggest the eventual breakdown of an initially mutualistic symbiosis. Evidence for convergent evolution of unrelated endosymbionts reflects adaptations to the intracellular environment of termite gut flagellates.

## INTRODUCTION

Bacterial endosymbionts of eukaryotes typically experience progressive genome erosion (1–4). Apart from several well-characterized endosymbionts in insect tissues, the absence of close but free-living relatives often impedes our understanding of the evolutionary processes and mechanisms underlying the transition from a host-associated to an intracellular lifestyle. A notable exception are the so-called Endomicrobia, which represent a family-level clade in the phylum *Elusimicrobiota* and have been detected almost exclusively in the intestinal tract of insects (5–7) and ruminants (7–9). The family *Endomicrobiaceae* comprises both free-living gut commensals of their respective hosts and closely related lineages of endosymbionts that abundantly colonize the cytoplasm of termite gut flagellates (10, 11). In the context of this study, we use the term “free-living” to distinguish host-associated bacteria that occur freely within the gut from those in an endosymbiotic association with protists.

All evolutionary “lower” termites (all families except Termitidae or “higher” termites) harbor diverse assemblages of flagellate protists that are essential for the digestion of lignocellulose (12, 13). The flagellates themselves are generally associated with bacterial symbionts that colonize the exterior surface, cytoplasm, or nucleus of their hosts (14–18). Although the symbionts belong to different bacterial phyla, they share conspicuous similarities in the metabolic capacities encoded by their respective genomes. Despite a substantial reduction in genome size, the biosynthetic pathways of the symbionts are mostly conserved, which led to the consensus that the symbionts either provide their flagellate host with fixed nitrogen and/or essential amino acids and cofactors (16, 19–21) or contribute metabolic capacities that facilitate cellulose degradation, such as reductive acetogenesis (19, 22). The shared presence of such traits suggests a parallel evolution of unrelated lineages of endosymbionts.

Although the diversity of *Endomicrobiaceae* in termite guts has been investigated in great detail and numerous associations with different termite gut flagellates have been documented (5–7, 11, 23, 24), it is unclear whether all intracellular symbionts were derived from a single endosymbiotic event or were independently acquired from different lineages of free-living ancestors. This is mostly caused by the poorly resolved phylogeny of *Endomicrobiaceae* in 16S rRNA-based studies (10, 11). Moreover, the family comprises only a single isolate, *Endomicrobium proavitum* (25), and a few endosymbionts with sequenced genomes (26–28). While the free-living *Endomicrobium proavitum* and the endosymbiotic *Candidatus* Endomicrobium trichonymphae share a purely fermentative metabolism, they differ in genome size and guanine-cytosine (GC) content and in their metabolic capacities, such as the uptake and activation mechanisms of their major energy substrates, suggesting that the substantial gene loss in the endosymbiont was accompanied by acquisition of novel functions (29). In the absence of genomic information on other representatives of the family, it remains open whether endosymbiosis in *Endomicrobiaceae* arose more than once and how free-living lineage(s) adapted to the intracellular lifestyle.

To address these questions, we reconstructed more than 1,700 bacterial genomes from gut metagenomes of 48 termite species, representing all major families, including all subfamilies of the flagellate-free Termitidae. This effort yielded 67 novel metagenome-assembled genomes (MAGs) of *Endomicrobiaceae*. Together with previously published genomes, including numerous MAGs from termites (30), ruminants (31–34), and anaerobic bioreactors (35) that had not been analyzed to date, this provided a total of 106 genomes. We used this comprehensive data set to analyze the phylogeny of *Endomicrobiaceae* and the metabolic potential of the individual lineages, with the aims of identifying the distribution of putative endosymbionts and tracing the loss and gain of relevant functions during the evolutionary history of *Endomicrobiaceae*.

## RESULTS

### Metagenome-assembled genomes

**Fig 1:**
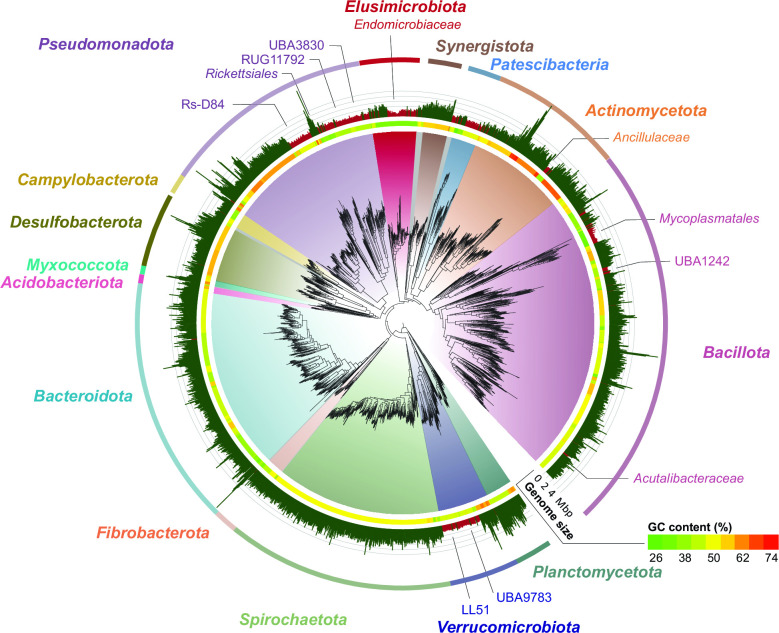
Phylogeny of 2,228 bacterial MAGs reconstructed from termite gut metagenomes. Phyla represented by more than 10 MAGs and subclades harboring putative endosymbionts are labeled. Bars illustrate genome size, MAGs < 1.5 Mbp are marked in red. GC content is indicated at family level. The maximum-likelihood tree was inferred from the concatenated amino acid alignment of 120 protein-coding genes generated by GTDB-Tk using the LG+F+I+G4 model of evolution. For a detailed classification of all MAGs and additional information, see Table S2.

Termite gut metagenomes yielded 1,723 MAGs of high and medium quality, ranging between 13 and 64 MAGs per host species (Table S1). Together with the 589 MAGs from our previous study (30), they comprise 2,228 MAGs from the bacterial domain and 84 MAGs from the archaeal domain. The archaeal MAGs comprised members of the euryarchaeal phyla *Thermoplasmatota* (33 MAGs), *Methanobacteriota* (15 MAGs), and *Halobacterota* (10 MAGs), and the crenarchaeal phylum *Thermoproteota* (26 MAGs); these have been covered in a separate study (36). The bacterial MAGs were classified into 30 phyla. The majority is represented by *Bacillota* (24.1%), *Bacteroidota* (15.6%), *Spirochaetota* (14.2%), and *Pseudomonadota* (13.3%). Most of the remaining MAGs fell into (in descending order) *Actinomycetota*, *Desulfobacterota*, *Verrucomicrobiota, Elusimicrobiota*, *Planctomycetota*, *Patescibacteria*, *Synergistota*, *Fibrobacterota*, *Campylobacterota*, *Acidobacteriota,* and *Myxococcota* (Fig. 1). The remaining 11 phyla were represented by less than 10 MAGs each (for details, see Table S2). Of particular interest for the present study were bacterial lineages comprising MAGs with small genome sizes (<1.5 Mbp) (Fig. 1). They include *Endomicrobiaceae* (*Elusimicrobiota*) and many lineages from other phyla that are already known to harbor endosymbionts of termite gut flagellates.

### Diversity of *Endomicrobiaceae*

**Fig 2:**
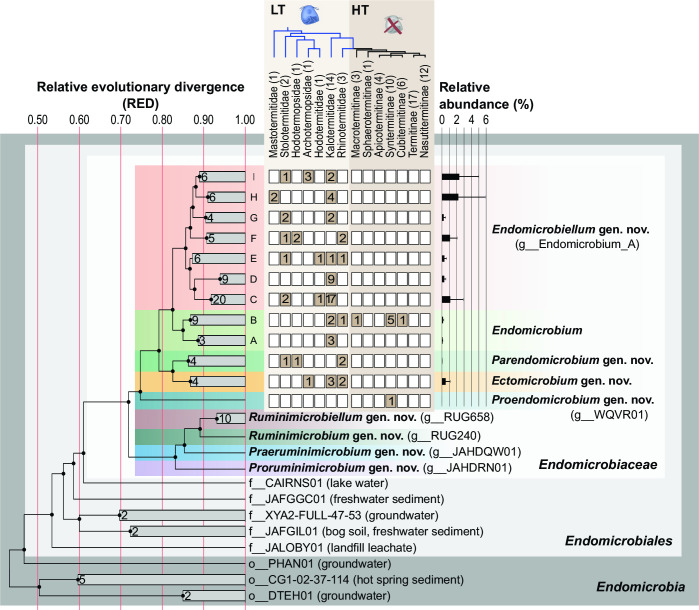
Phylogeny of the class *Endomicrobia*, illustrating the newly proposed taxonomy of *Endomicrobiaceae*. Provisional names from the GTDB taxonomy and environmental origin of ancestral lineages are indicated. The number of MAGs recovered from different families of lower termites (LT) and subfamilies of higher termites (HT), and the relative abundance (average) of the MAGs among the total sequencing reads of the respective metagenomes are shown. The maximum-likelihood tree was inferred using the same parameters as in Fig. 1 and was normalized using relative evolutionary divergence (RED) values determined with PhyloRank. Bullets on internal nodes indicate SH-aLRT/UFBoot support (⚫, both ≥ 95/99%; 1,000 replicates each). The number of genomes in the collapsed clades is indicated (for details, see Fig. 3; Fig. S1).

Almost all MAGs from the phylum *Elusimicrobiota* were members of the family *Endomicrobiaceae*. A detailed phylogenomic analysis that included all genomes of this family available to date (Table S3) revealed two highly supported monophyletic clades, one consisting exclusively of representatives from termites, the other of MAGs recovered from various ruminants or an anaerobic digester (Fig. 2).

**Fig 3:**
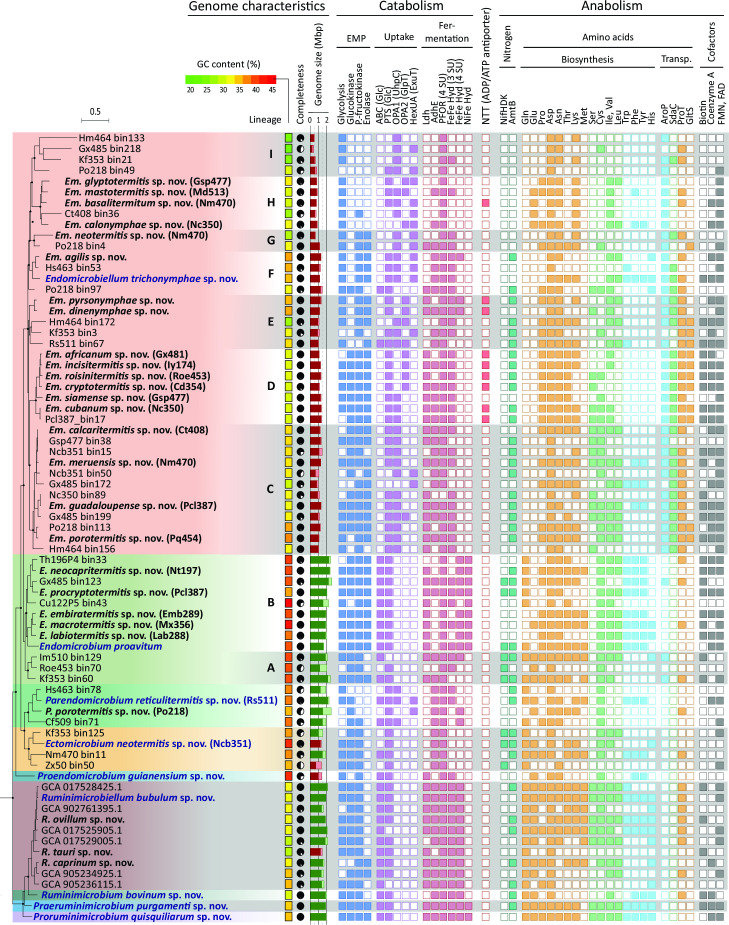
Expanded phylogeny of *Endomicrobiaceae*, illustrating the key genes/pathways encoded by representative genomes of each lineage. The tree is the same as in Fig. 2; type strains are indicated in bold (type species in blue). More details, including the accession numbers of all genomes and the position of additional, low-quality MAGs, are given in Fig. S2. Genome size was estimated from assembly size and completeness (indicated by shaded and filled bars). Glycolysis was considered absent if fructose-bisphosphate aldolase or pyruvate kinase is missing; the absence of other enzymes of the Embden-Meyerhoff pathway (EMP) is discussed in the text. HexUA, hexuronate; Ldh, lactate dehydrogenase; AdhE, bifunctional aldehyde–alcohol dehydrogenase; PFOR, pyruvate:ferredoxin/flavodoxin oxidoreductase; Pta, phosphate acetyltransferase; Ack, acetate kinase; Hyd, hydrogenase; NifHDK, nitrogenase; AmtB, ammonium transporter; AroP, aromatic amino acid transporter; SdaC, serine transporter; ProT, proline transporter; GltS, sodium–glutamate symporter. For information on the individual genes of each pathway, including xylose and hexuronic acid metabolism and components of the cell envelope, see Fig. S4, S10, S12 and S13.

Based on tree topology and relative evolutionary divergence (RED), the termite-specific clade comprises the genus *Endomicrobium* and four so-far unclassified genus-level lineages without cultured representatives. We propose to classify the MAGs in these lineages in the new genera *Proendomicrobium*, *Ectomicrobium*, *Parendomicrobium*, and *Endomicrobiellum* (see “Taxonomy” below). Almost all genera, including also the well-supported subgroups (A–I) of *Endomicrobium* and *Endomicrobiellum*, are represented by at least one high-quality MAG (completeness ≥ 90%, contamination ≤ 5%). All previously characterized endosymbionts, including all candidate species described to date (*Ca*. Endomicrobium trichonymphae, *Ca*. Endomicrobium pyrsonymphae, *Ca*. Endomicrobium agile, and *Ca*. Endomicrobium dinenymphae) fall into the new genus *Endomicrobiellum*. The second clade comprises four so-far unclassified genus-level lineages, two of which are represented by MAGs from the rumen. We propose to classify the MAGs in these lineages in the new genera *Proruminimicrobium*, *Praeruminimicrobium*, *Ruminimicrobium*, and *Ruminimicrobiellum* (see “Taxonomy” below).

The vast majority of the *Endomicrobiaceae* MAGs recovered from termite guts were from lower termites (Fig. 2). Most of them fell into the genus *Endomicrobiellum*. Except for *Prorhinotermes canalifrons*, each termite sample yielded one or more medium- or high-quality MAGs. Only a few MAGs were recovered from higher termites. One fell into the genus *Proendomicrobium*, all others into the genus *Endomicrobium*, which are of low relative abundance in both lower and higher termites (Fig. 2). Each MAG represents a phylotype unique to a particular termite (Table S3). Low-quality MAGs were recovered from many samples, indicating the presence of additional, albeit smaller populations. The inclusion of low-quality MAGs in the phylogenomic tree corroborated the status of *Proendomicrobium* (represented by only a single medium-quality MAG) as a separate lineage (Fig. S1). A 16S rRNA-based analysis of *Endomicrobiaceae*, including all homologs recovered from the MAGs and the corresponding metagenomes, allowed us to identify all genus-level lineages defined in the phylogenomic tree except *Proendomicrobium* (Fig. S3). This includes the genus *Parendomicrobium*, which comprises MAGs that are linked to ectosymbionts of a *Spirotrichonympha*-like flagellate in *Porotermes adamsoni* (24) and to the gut microbiota of *Reticulitermes flavipes* by several unbinned sequences from the corresponding metagenomes.

Although most genera of *Endomicrobiaceae* comprise representatives with relatively large genomes (average size estimates 2.11 Mbp ± 0.26 Mbp) and a relatively high GC content (average 38.0% ± 3.2%), the genomes of *Proendomicrobium, Ectomicrobium*, and *Endomicrobiellum* are considerably smaller (average 1.16 Mbp ± 0.30 Mbp; Table S4). Members of the genus *Endomicrobiellum*, which comprises all established endosymbionts and is absent from higher termites, have the smallest genomes (average 1.13 Mbp ± 0.29 Mbp) and lowest GC contents (average 32.3% ± 3.0%) of the entire family, corroborating that they are endosymbionts of termite gut flagellates.

### Energy substrates

**Fig 4:**
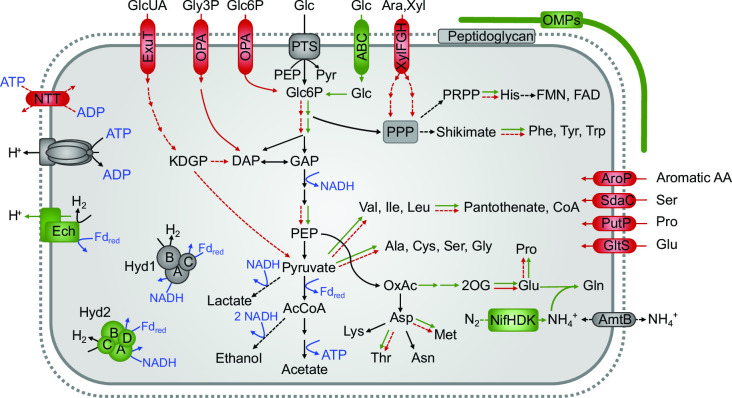
Metabolic capacities shared by members of *Endomicrobiaceae*. Pathways present only in putatively free-living lineages (green) or in endosymbionts of the genus *Endomicrobiellum* (red) are highlighted. Dashed lines indicate that a pathway is not present in all members of each group. GlcUA, glucuronic acid; Gly3P, glycerol 3-phosphate; PTS, phosphotransferase system; ABC, ABC transporter; XylFGH, arabinose/xylose transporter; OMPs, outer membrane proteins; NTT, nucleotide triphosphate transporter; PPP, pentose phosphate pathway, PRPP, phosphoribosyl pyrophosphate; KDGP, 2-keto-3-deoxygluconate 6-phosphate; for more abbreviations, see the legend of Fig. 3.

We compared the pathways involved in energy metabolism and assimilatory functions encoded in the genomes of *Endomicrobiaceae* (Fig. 3). The metabolic capacities shared by all family members and major differences between the putatively free-living lineages and endosymbiotic genus *Endomicrobiellum* are summarized in Fig. 4.

**Fig 5:**
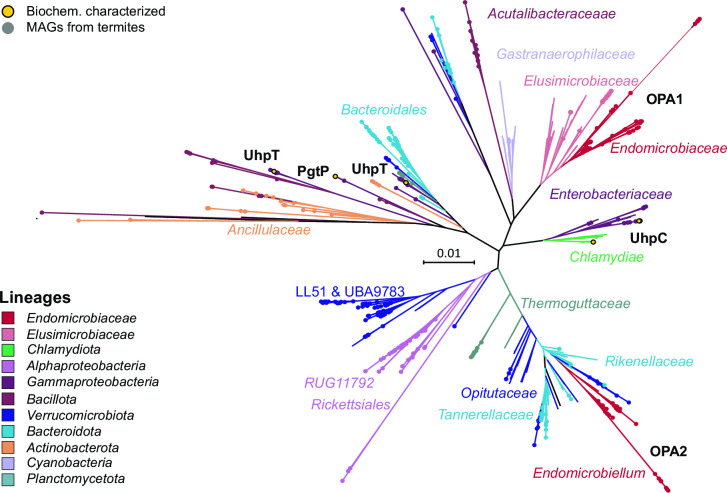
Phylogeny of the OPA family transporters in *Endomicrobiaceae* and their homologs in public databases. Organismal lineages are color coded; filled circles indicate homologs in MAGs obtained from termite guts. Biochemically characterized homologs (SWISS-PROT) are highlighted. The maximum-likelihood tree is based on a curated alignment of 428 amino acid positions and was inferred under the LG+F+R9 model of evolution. Bootstrap values were omitted for clarity.

All members of *Endomicrobiaceae* are obligately anaerobic sugar fermenters. They generally possess two glucose uptake systems: a putative ABC transporter of the CUT1 family and a phosphotransferase system (PTS) of the mannose type. Although the ABC transporter was lost in all members of *Endomicrobiellum*, the PTS system is preserved in most lineages. Instead, almost all *Parendomicrobium* and *Endomicrobiellum* genomes encode one or two transporters of the organophosphate:inorganic phosphate antiporters (OPA) family, which are absent from all other genera. They fall into the radiation of sugar phosphate transporters from a wide range of bacterial lineages, including the biochemically characterized glucose-6-phosphate (Glc6P) transporter in *Chlamydia pneumoniae* and Glc6P-transporting sensor of *Escherichia coli* (UhpC) (37) (Fig. 5). One clade (OPA1) clusters among homologs from *Elusimicrobiaceae,* the other (OPA2) clusters among homologs of *Bacteroidales* and *Opitutaceae*, which are abundantly represented among the MAGs of this study. Homologs from this clade are frequently annotated as glycerol-3-phosphate (Gly3P) transporters, but because none of the transporters in the respective clades have been biochemically characterized, their substrate specificity remains tentative. Nevertheless, the results suggest that members of the genera *Parendomicrobium* and *Endomicrobiellum* have acquired the capacity to take up sugar phosphates.

**Fig 6:**
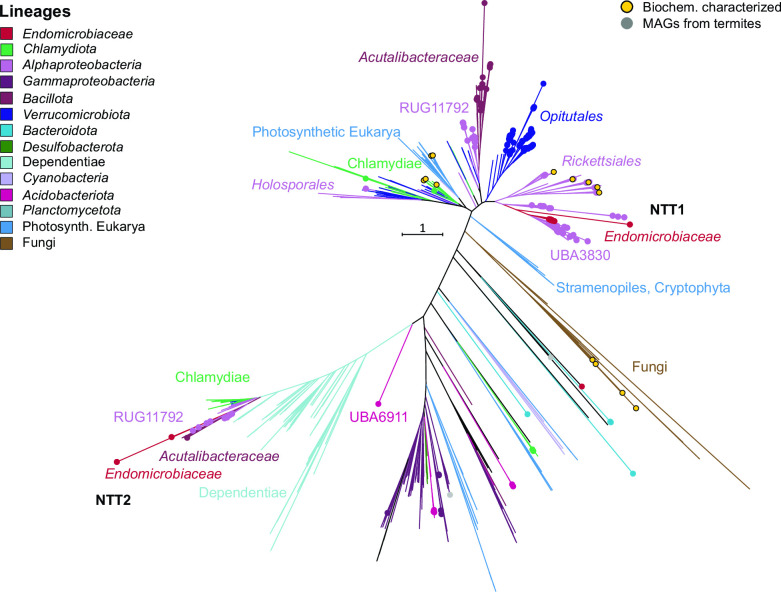
Phylogeny of the NTT in *Endomicrobiaceae* and their homologs in public databases. Organismal lineages are color coded; filled circles indicate homologs in MAGs obtained from termite guts. Biochemically characterized homologs (SWISS-PROT) are highlighted. The maximum-likelihood tree is based on a curated alignment of 982 amino acid positions and was inferred under the LG+F+R5 model of evolution. Bootstrap values were omitted for clarity.

Intriguingly, members of *Endomicrobiellum* lineage D encode a nucleotide triphosphate transporter (NTT) that is embedded among homologs from the order UBA3830, a lineage of uncultured alphaproteobacteria from termites and ruminants abundantly represented among the MAGs of this study, and that is closely related to biochemically characterized ATP/ADP antiporters of *Rickettsiales* (38) (Fig. 6). The residues critical for function and substrate specificity in the antiporter of *Arabidopsis thaliana* (39) are conserved in both the homologs of *Endomicrobiellum* lineage D and the order UBA3830 (Fig. S4). A few MAGs from other *Endomicrobiellum* lineages encode NTTs that fall into the radiation of homologs present in other MAGs from termite guts (*Acutalibacteraceae* and the alphaproteobacterial order RUG11792).

### Fermentative metabolism

All *Endomicrobiaceae* are strict anaerobes that convert glucose to pyruvate via the canonical Embden-Meyerhoff pathway (EMP; Fig. 3). There is no evidence for respiratory metabolism, and a catalase gene is absent from all genomes. However, genes encoding key enzymes of the glycolytic pathway are absent in many members of *Endomicrobiellum* and several other lineages. The loss of glucokinase (Glck) is compensated by the PTS and/or the acquisition of UhpC, both of which provide the EMP with glucose 6-phosphate (see "Energy substrates"). While the formation of fructose 1,6-bisphosphate via pyrophosphate–fructose 6-phosphate 1-phosphotransferase (Pfp) instead of the canonical phosphofructokinase (Pfk) would even save ATP (40), the loss of enolase is difficult to explain. Notably, an enolase gene is consistently absent not only in two apical clades of *Endomicrobiellum* (lineages H–I) but also from members of other genera (e.g., *Proendomicrobium*, *Parendomicrobium*, and *Ruminimicrobiellum*). Considering that a methylglyoxal synthase gene is absent in *Endomicrobiaceae*, the enolase reaction cannot be circumvented via the methylglyoxal pathway, i.e., the conversion of dihydroxyacetone phosphate (DAP) to pyruvate (41).

Several subclades of *Endomicrobiellum* and members of the genera *Ectomicrobium* and *Parendomicrobium* possess gene sets for the uptake and metabolism of hexuronic acids via the 2-keto-3-deoxy-phosphogluconate (KDPG) pathway (Fig. 3). Genes for the metabolism of xylose and arabinose, which had been detected already in single-cell amplified genomes of *Endomicrobiellum dinenymphae* and *Endomicrobiellum pyrsonymphae* (28), were found also in a few MAGs from lineages C and G (Fig. S5). Genes for glucose-6-phosphate dehydrogenase and 6-phosphogluconate dehydratase, key enzymes of the Entner-Doudoroff pathway, are absent from the entire family.

*Endomicrobiaceae* generally oxidize pyruvate to acetyl-CoA via the canonical four-subunit pyruvate:ferredoxin oxidoreductase (PFOR) present in many anaerobes. Only *Endomicrobium proavitum* and members of *Endomicrobium* subcluster A possess a flavodoxin-dependent homolog with fused subunits, which was apparently acquired from *Elusimicrobiaceae* ([27]; Fig. S5 and S6). In all members of the termite clade, acetyl-CoA is converted to acetate by phosphotransacetylase (Pta) and acetate kinase (Ack). Pta is absent in the entire ruminant clade, whereas an acetyl-CoA synthetase (AMP-forming) is present in most *Endomicrobiaceae* (Fig. S5).

Reduced coenzymes (NADH and ferredoxin) are reoxidized either by the reduction of pyruvate to lactate and/or ethanol, or by the formation of hydrogen. While the lactate dehydrogenase (Ldh) of *Endomicrobiaceae* falls into the radiation of homologs from other *Elusimicrobiota* (Fig. S7), the homologs of the bifunctional aldehyde/alcohol dehydrogenase (AdhE) in the genera *Ectomicrobium*, *Parendomicrobium*, and *Endomicrobiellum* are not directly related and were apparently acquired independently via horizontal gene transfer from other phyla (Fig. S8).

Hydrogenase genes of *Endomicrobiaceae* were previously identified in *Endomicrobium proavitum* and *Endomicrobiellum trichonymphae* (29). Members of most genera encode a trimeric, electron-bifurcating [FeFe] hydrogenase (HydABC) of group A3 that is absent from other representatives of *Elusimicrobiota*. Several free-living lineages, including those from ruminants, possess a second, tetrameric homolog of the same subclass but of independent phylogenetic origin (Fig. S9). A membrane-bound [NiFe] hydrogenase of group 4g, which comprises uncharacterized, presumably ferredoxin-dependent, hydrogen-evolving enzymes (42), is restricted to members of the genus *Endomicrobium* and only distantly related to homologs from the order *Elusimicrobiales* (see Fig. S10).

### Biosynthetic pathways

*Endomicrobiaceae* generally lack genes encoding fructose 1,6-bisphosphatase (Fbp) and PEP carboxykinase (PckA), the key enzymes of gluconeogenesis from pyruvate. The pathway for the synthesis of oxaloacetate, which provides the carbon skeleton for amino acids of the aspartate family, including the corresponding aminotransferases, is present in all *Endomicrobiaceae*. The pathway for the synthesis of 2-oxoglutarate, the precursor for amino acids of the glutamate family, however, is absent from most members of *Endomicrobiellum* (Fig. S11). Most members of the free-living genera encode an ammonia transporter (AmtB), glutamine:oxoglutarate aminotransferase (GltA), and glutamine synthetase (GlnA), the enzymes required for ammonia assimilation into glutamine. *Endomicrobiaceae* lack a canonical citrate synthase, but all genera, including several lineages of the endosymbiotic *Endomicrobiellum*, encode homologs of a putative *Re*-type citrate synthase, which fall into the same clade as the biochemically characterized enzymes from other phyla (29).

All putatively free-living lineages possess pathways for the biosynthesis of almost all proteinogenic amino acids (Fig. 4; Fig. S11). Glutamate is synthesized by a tetrameric variant of GltA that is present also in *Pyrococcus* sp. (43). An asparagine synthetase (AsnAB) is absent from all *Endomicrobiaceae*, but the majority of the MAGs encode an unusual aspartyl-tRNA synthetase (AspS2), which loads an aspartyl residue onto tRNA^Asn^, where it is subsequently converted to an asparaginyl residue by aspartyl-tRNA amidotransferase (GatCAB) (44). All *Endomicrobiaceae* synthesize glycine from serine via serine hydroxymethyltransferase (GlyA); the alternative threonine utilization pathway is absent. These findings explain the prototrophy of *Endomicrobium proavitum* for all amino acids (29) except serine. A complete phosphoserine pathway (SerABC) is present only in members of the ruminant clade. Members of the termite clade encode only the 3-phosphoglycerate dehydrogenase (SerA) of the phosphoserine pathway and the serine:pyruvate aminotransferase (Spa) but not the hydroxypyruvate reductase (Hpr) of the glycerate pathway.

Despite massive genome erosion in the genus *Endomicrobiellum*, several lineages retained pathways for the biosynthesis of up to 15 amino acids (Fig. 3). Although pathways for the biosynthesis of aspartate, asparagine, lysine, glycine, and alanine are conserved in almost all endosymbionts, the capacity to synthesize serine, glutamine, and aromatic amino acids was lost in several subclades. GltA is present in some lineages, but the oxidative branch of the tricarboxylic acid (TCA) cycle forming 2-oxoglutarate is missing in all members of the genus. The presence of several amino acid transporters, such as an aromatic amino acid transporter (AroP), a serine transporter (SdaC), a proline symporter (ProT), and a glutamate symporter (GltS), exclusively in members of the genus *Endomicrobiellum* suggests that the loss of the respective biosynthetic capacities is compensated by the uptake of amino acids provided by their flagellate hosts.

A few members of the genera *Ectomicrobium* and *Endomicrobium,* including the diazotrophic *Endomicrobium proavitum* (25), possess a gene cluster encoding a group IV nitrogenase (NifDKH) (Fig. 3), which is represented also in *nifH* gene inventories of termite guts (e.g., references 45, 46; Fig. S12). The conservation of AmtB in many *Endomicrobiellum* lineages may indicate the necessity to export NH_4_^+^ resulting from a utilization of amino acids as alternative carbon sources (see Discussion).

Most representatives of *Endomicrobiaceae* possess the biosynthetic pathways for most cofactors (Fig. 3). Although the capacity to synthesize biotin from pimelyl-CoA is present in most genera, the synthesis of the pimelate moiety is unclear and (as in many other bacteria) differs from the established pathways (47). Several subclades of *Endomicrobiellum* (lineages D, G–I) lost the ability to synthesize biotin, coenzyme A, and flavin nucleotides (FMN, FAD) (Fig. S11).

The pathways for the biosynthesis of peptidoglycan are conserved in most members of *Endomicrobiaceae*, suggesting that also the endosymbiotic lineages have retained the capacity to produce a murein sacculus (Fig. S13). Notably, the bifunctional murein transglycosylase/transpeptidase (MrcB) is absent from almost the entire termite clade, and a d-alanine:d-alanine ligase (Ddl) is lacking in several subclades of *Endomicrobiellum*. Also, key genes for lipopolysaccharide (LPS) synthesis and several outer membrane proteins, including OmpA, TolB, and the vitamin B_12_ transporter BtuB, are present in most genera but absent in many members of *Endomicrobiellum*. Components characteristic for the outer membrane of other bacteria (e.g., the outer membrane complex assembly proteins BamBCD, the long-chain fatty acid protein FadL, and the LPS assembly protein LptD) are absent from all *Endomicrobiaceae*.

The only cell appendages encoded by *Endomicrobiaceae* are pilins. The absence of a flagellar machinery from all genomes of the phylum *Elusimicrobiota* is consistent with previous studies (26, 29, 48) and agrees with the observation that all isolates from the phylum *Elusimicrobiota* are non-motile (49).

### Genes for informational processing, genome modification, and repair

We found no notable difference between putatively free-living and endosymbiotic lineages in gene functions that are typically lost during genome erosion in bacterial endosymbionts of insect tissues, such as DNA replication or repair, protein folding and stability, and competence and transformation (50) (Fig. S14). Transposons were detected in more than half of the genomes but seem to be randomly distributed among free-living and endosymbiotic lineages (Table S5). CRISPR-Cas systems were present only in a few representatives (15 MAGs) of diverse lineages, including 11 systems of type II-C and 3 systems of type I-C (Table S6). Their random distribution between free-living and endosymbiotic lineages makes it unlikely that these subgroups are involved in genome reduction and rearrangement in *Endomicrobiellum* (27). Also, restriction-modification systems, which are abundant in *Endomicrobiellum trichonymphae* and have been implicated in genome rearrangement (51), are rare and randomly distributed among *Endomicrobiaceae* (Table S6).

### Potential endosymbionts in other phyla

Additionally, numerous lineages of MAGs from other bacterial phyla that occur exclusively in lower termites showed genome sizes that were in the same range as those of the genus *Endomicrobiellum* (<1.5 Mbp) (Fig. 1). Several lineages represented established endosymbionts of termite gut flagellates (“*Ancillulaceae*” and *Acutalibacteraceae*) and other protists (e.g., *Rickettsiales*, *Acholeplasmatales*, *Mycoplasmatales*, and the *Opitutales* families LL51 and UBA9783). Others may represent so-far unknown lineages of endosymbionts (e.g., the alphaproteobacterial orders RUG11792 and UBA3830). Additional lineages with small genomes were represented by MAGs from both lower termites and (flagellate-free) higher termites (e.g., *Patescibacteria, Dehalococcoidales*, the clostridial order UBA1242, and the alphaproteobacterial order Rs-D84).

**Fig 7:**
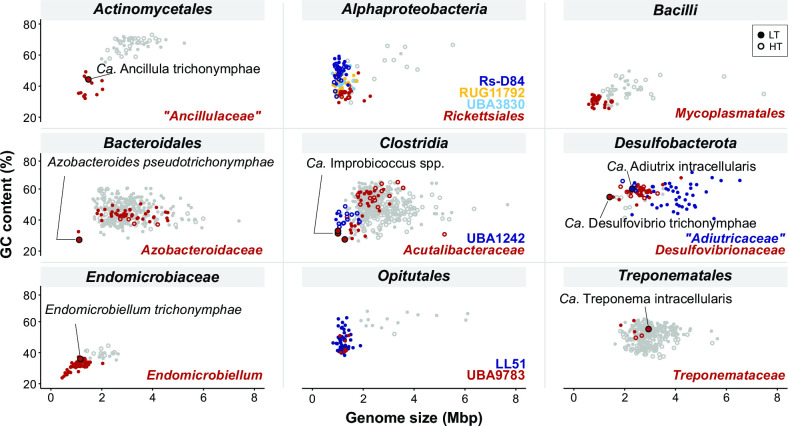
Genome size and GC content of the MAGs from termite guts, comparing lineages that comprise established and putative endosymbionts (in color) with their closest relatives (in gray). The origin of the MAGs from lower termites (⚫) and higher termites (〇) is indicated; the genomes of well-characterized endosymbionts are marked. For an interactive map that shows the identity of each MAG, see Fig. S15.

Genome size and GC content of “*Ancillulaceae*,” three clades of *Alphaproteobacteria* (*Rickettsiales*, UBA3830, and RUG11792), and a large clade of *Verrucomicrobiota* (families LL51 and UBA9783) were consistently lower than those of MAGs from the corresponding parent taxa (Fig. 7). Other lineages comprised only a few MAGs with significantly smaller genomes (e.g., “*Adiutricaceae*,” *Acutalibacteraceae*, *Azobacteroidaceae*, and *Desulfovibrionaceae*) and/or consisted of MAGs from both lower and higher termites (e.g., UBA1242, Rs-D84, “*Adiutricaceae*”), corroborating the notion that not all MAGs of these lineages represent endosymbionts of termite gut flagellates (19, 52, 53).

An inspection of all MAGs representing putative endosymbionts for symbiosis-related traits detected in *Endomicrobiellum* revealed that members of almost all lineages have organophosphate transporters (OPAs) (Table S7). KDPG aldolase and the corresponding transporter for hexuronic acids were present only in the already established endosymbionts among *Azobacteroidaceae*, “*Ancillulaceae*,” and *Acutalibacteraceae*. An NTT is present in the endosymbiotic (or putatively endosymbiotic) *Acutalibacteraceae*, *Opitutales*, and the alphaproteobacterial orders UBA3830 and RUG11792. Phylogenetic analyses of the key genes for the respective functions revealed that they are typically absent from related taxa from other environments and most likely acquired by horizontal gene transfer within the termite gut (Fig. 5 and 6; Fig. S16 and S17). Only a few lineages of flagellate endosymbionts (“*Adiutricaceae*,” “*Ancillulaceae*”) encode homologs of aromatic amino acid transporters (AroP), which were most likely derived from distantly related organisms (typically from different phyla) (Fig. S17). The capacity for nitrogen fixation is present only among “*Adiutricaceae*,” *Azobacteroidaceae*, and *Desulfovibrionaceae* (Table S7).

## DISCUSSION

The discovery of the first sequences of the “Termite group 1” (54, 55) and their association with termite gut flagellates (5, 7), the phylogenetic origin of Endomicrobia, and the relationship between free-living and endosymbiotic lineages remained unresolved. Our study revealed that host-associated *Endomicrobiaceae* originated among aquatic ancestors that subsequently colonized the intestinal tract of insects and ruminants. Although most lineages represent free-living gut commensals, a common ancestor of the genus *Endomicrobiellum* engaged in an intracellular symbiosis with termite gut flagellates. Progressive genome reduction led to a breakdown of glycolysis and many biosynthetic pathways, which was compensated by the acquisition of new functions via horizontal gene transfer. In some subgroups, the apparent loss of all biosynthetic capacities that could potentially benefit the flagellate host indicates the decline of an originally mutualistic relationship.

### Phylogenetic diversity of *Endomicrobiaceae*

The presence of two lineages from anaerobic digestors (*Proruminimicrobium* and *Praeruminimicrobium*) in a position ancestral to the host-associated members of the ruminant clade (*Ruminimicrobium* and *Ruminimicrobiellum*) corroborates the origin of the family *Endomicrobiaceae* among lineages of aquatic bacteria in the order *Endomicrobiales* (49). Although the environmental origin of the ruminant clade is supported also by 16S rRNA-based diversity data, the origin of the termite-associated clade remains unresolved (Fig. S3). Previous 16S-rRNA-based studies detected members of *Endomicrobiaceae* also in other insects, such as cockroaches (7, 10, 11) and a scarab beetle larva (56), but, with the exception of lower termites, always in low abundance (11, 57, 58). This explains the absence of *Endomicrobiaceae* among MAGs from cockroaches (59, 60) and the low recovery from higher termites (Fig. 2). Notably, the sister position of the 16S rRNA genes of *Endomicrobiaceae* from cockroaches to the genus *Endomicrobium* is only poorly supported (Fig. S3) and remains to be verified with phylogenomic data. The 16S rRNA gene tree documents an affinity of *Ca*. Endomicrobium superficiale, the ectosymbionts of *Trichonympha magna* from *Porotermes adamsoni*, and spirotrichosomid flagellates from the related *Stolotermes victoriensis* (24), to the genus *Ectomicrobium*, but in the absence of a genome sequence, this assignment must remain tentative.

The genus *Endomicrobiellum* comprises all endosymbiotic representatives that were localized in previous studies (5–7, 23, 61). Compared to other genera of *Endomicrobiaceae*, their genomes are strongly reduced, which is in agreement with the endosymbiotic nature of the entire genus. Notably, also members of the genus *Ectomicrobium*, which are associated with gut flagellates (24), have slightly smaller genomes than most other family members, including their sister genus, *Parendomicrobium* (Table S4).

The genera *Ruminimicrobium* and *Ruminimicrobiellum* comprise MAGs recovered from large-scale studies of ruminal microbiota of sheep, cows, deer, and goats (31–34, 62). It has been speculated that *Endomicrobiaceae* present in the rumen are associated with ciliates (9). This is supported by a significantly smaller genome size in members of the genus *Ruminimicrobiellum* compared to most other lineages of the family (Table S4), and by our observation that 16S rRNA genes of *Endomicrobiaceae* recovered from capillary-picked suspension of rumen ciliates (*Isotricha* spp.) fall into the radiation of this genus (U. S. Mies and A. Brune, unpublished data; see Fig. S3). Therefore, it is possible that an association with protists has evolved independently in three lineages of *Endomicrobiaceae*.

### Genome erosion and acquisition of new functions in *Endomicrobiellum*

The fermentative energy metabolism of *Endomicrobium proavitum* (29) is shared by all members of *Endomicrobiaceae*. Unlike several other lineages of the class *Elusimicrobia* that encode respiratory chains for oxygen and other inorganic electron acceptors (63), all members of *Endomicrobiaceae* are strict anaerobes that activate glucose to Glc6P with glucokinase and/or a PTS, and ferment Glc6P via the EMP pathway to ethanol, lactate, acetate, hydrogen, and CO_2_. All members of the endosymbiotic genus *Endomicrobiellum*, including the previously characterized *Em. trichonymphae* (26, 27, 29), lack the ABC transporter and often also the PTS for the uptake of glucose. These gene losses are compensated by two organophosphate:phosphate antiporters (OPA1 and OPA2) that were independently acquired by different subclades and allow the direct uptake of sugar phosphates from the cytoplasm of their respective hosts.

Although annotated as Glc6P and Gly3P transporters, both OPA1 and OPA2 fall into phylogenetic clades without biochemically characterized representatives (Fig. 5); therefore, their exact substrates remain uncertain. Nevertheless, either of these substrates would serve to link the energy metabolism of endosymbiont and host, as observed in other bacteria with an intracellular lifestyle (e.g., *Listeria* and *Chlamydia* [64, 65]). Here, the switch to sugar phosphates is considered an adaptation to the low concentrations of free glucose in eukaryotic cells that provides an additional, energetic advantage because no ATP is needed for substrate activation. It is not clear whether the acquisition of OPAs by members of the genus *Endomicrobiellum* was a predisposition for an intracellular lifestyle, as in the ancestors of *Chlamydia* (65), or a response to the conditions in their intracellular habitat. In any case, these transporters may be the only means to obtain an energy substrate for those members of the genus *Endomicrobiellum* that lack both glucokinase and PTS (lineages H and I).

The absence of enolase in several basal lineages with an otherwise complete glycolytic pathway is rather perplexing. The same situation has been reported for a variety of host-associated bacteria, including *Treponematales* and *Bacteroidales* symbionts of termite gut flagellates (22, 66) and several *Saccharibacteria* (67, 68) and *Clostridiales* from mammals (69). Because a methylglyoxylate shunt, which allows circumvention of the enolase function in the rumen bacterium *Butyrivibrio proteoclasticus* (70), is absent from all *Endomicrobiaceae*, it remains unclear how members of these lineages generate the phosphoenolpyruvate required, e.g., by PTS.

The fermentative metabolism of glucose and other energy substrates requires regeneration of the reduced cofactors formed in the oxidative part of the pathway. Although most members of *Endomicrobiaceae* encode homologs of Ldh and/or AdhE and at least one hydrogenase, the lineages of *Endomicrobiellum* without a functional glycolysis (lineages H and I) also lack the enzymes that reoxidize NADH and reduced ferredoxin, indicating that they lost the capacity for sugar fermentation. It is possible that they conserve energy by oxidative decarboxylation of amino-acid-derived 2-oxoacids to the corresponding acyl-CoA esters, as in *Elusimicrobium minutum* (71) and in hyperthermophilic archaea (72, 73).

An interesting case is presented by the MAGs that possess an ATP/ADP antiporter, which enables them to import ATP directly from the host. This NTT was independently acquired from uncultured alphaproteobacteria (orders UBA3830 and RUG11792) by *Endomicrobiellum* lineage D and several MAGs from other lineages. ATP/ADP antiporters and other NTTs are regarded as a hallmark of parasitism and are common in intracellular parasites of amoebae, such as *Chlamydia* and *Rickettsia*, where they are used by the parasites to obtain energy and nucleotide triphosphates for their growth and replication from their respective hosts (65, 74). However, NTTs are found also in free-living, non-parasitic bacteria (75); therefore, it remains open to speculation whether members of *Endomicrobiellum* have evolved into energy parasites or still provide a function that is beneficial for their flagellate hosts.

### Host–symbiont interactions

The intracellular location provides endosymbionts with a protective barrier against environmental forces. At the same time, an intact cell envelope may also hinder horizontal gene transfer and the exchange of metabolites between symbiont and host, which explains why the pathways for the synthesis of peptidoglycan and an outer membrane are frequently lost during genome erosion (76). Members of the genera *Endomicrobiellum* retained the capacity to synthesize a murein sacculus, LPS, and several outer membrane proteins (OMPs), which suggests that their cell envelope is still intact. However, the absence of the bifunctional transglycosylase/transpeptidase (MrcB) and d-alanine:d-alanine ligase (Ddl) in several subclades of *Endomicrobiellum* may affect the integrity of their cell wall (77), which may facilitate metabolite exchange between endosymbionts and host.

By default, an intracellular bacterium must synthesize all amino acids and vitamins that it cannot acquire from its host. Therefore, any deleterious mutations in its biosynthetic machinery must be compensated by an uptake mechanism that was either present already in the free-living ancestors of the endosymbiont or was later acquired by horizontal gene transfer. These two scenarios are exemplified by the ancestral presence of an uptake system for a proline transporter (ProT) and the acquisition from other bacteria of transporters for serine (SdaC), glutamate (GltS), and aromatic amino acid (AroP). These latter transporters are present in almost all lineages of *Endomicrobiellum* and are most closely related to homologs from unrelated bacteria present in termite guts.

The acquisition of amino acid transporters and conservation of many biosynthetic pathways in the genus *Endomicrobiellum* are in agreement with the long-standing hypothesis that flagellate endosymbionts provide essential amino acids to their respective hosts. Hongoh et al. (26) found that despite the severe genome erosion of *Em. trichonymphae*, pathways for the biosynthesis of aromatic amino acids are mostly conserved and several key genes are duplicated, which suggests that the endosymbionts provide the flagellate with aromatic amino acids. However, the capacity to synthesize tryptophane, tyrosine, and phenylalanine is not conserved in all lineages of the genus *Endomicrobiellum*. The same is true for biosynthetic pathways for proline and histidine. All members of the genus lack glutamine synthetase (GlnA), indicating a general dependence of the endosymbionts on the provision of glutamine by the host cell. It is unclear how this is accomplished because all members of *Endomicrobiellum* lack a glutamine transporter (GlnT). Lineage I lost the capacity to synthesize almost all amino acids, suggesting that they have lost their original function and may no longer play an essential role in the symbiosis. This applies also to a putative role of the endosymbionts in the provision of specific vitamins because the distribution of the corresponding biosynthetic pathways among different lineages is not conclusive (Fig. 3).

A function that is frequently encountered among bacterial symbionts of eukaryotic hosts is dinitrogen fixation. Examples among termite gut flagellates are the endosymbiotic *Azobacteroides pseudotrichonymphae* (78) and flagellate-associated bacteria in the gut of dry-wood termites (45, 79). Unlike the homolog of nitrogenase group VI, which occurs in other lineages of the phylum *Elusimicrobiota* and is potentially involved in tetrapyrrole modification (63), the group IV homologs encountered in members of the genera *Ectomicrobium* and *Endomicrobium* were shown to encode a functional nitrogenase (25). All other lineages of *Endomicrobiaceae*, including the genus *Endomicrobiellum*, lack nitrogenase and cannot contribute to dinitrogen fixation.

### Horizontal gene transfer among flagellate symbionts drives convergent evolution

There is abundant evidence supporting a convergent evolution among the endosymbionts of termite gut flagellates. Significant genome reduction has been reported for endosymbiotic representatives of *Acutalibacteraceae* (53), “*Ancillulaceae*” (21), *Azobacteroidaceae* (78), “*Adiutricaceae*” (19), and *Treponemataceae* (22). The same trend seems to be present also among the putatively endosymbiotic members of *Rickettsiales* and related *Alphaproteobacteria* (orders RUG11792 and UBA3830), *Mycoplasmatales*, and *Opitutales* (families LL51 and UBA9783) (Fig. 7). Notably, endosymbiotic representatives of these lineages not only co-occur in the same termites but also often co-localize in the same flagellate species, which provides ample opportunity for horizontal gene transfer.

Horizontal gene transfer among flagellate symbionts is most evident in the case of sugar phosphate transporters (OPAs), which are present in almost all lineages of established and putative flagellate endosymbionts except intracellular treponemes (Table S7). One homolog (OPA2) of *Endomicrobiellum* clusters among a clade of OPAs from termite-associated MAGs; the other homolog (OPA1) has close relatives among distantly related *Elusimicrobiaceae* associated with arthropods and ruminants but is absent from all other lineages of the *Elusimicrobiota* phylum. Their placement among the radiation of homologs from termite gut MAGs of other phyla suggests that both *Endomicrobiaceae* and *Elusimicrobiaceae* acquired their OPAs independently of each other from the same donor.

Another case of convergent evolution involves the nucleotide antiporters (NTTs), which were independently acquired by *Endomicrobiellum* from putative flagellate endosymbionts in the alphaproteobacterial orders UBA3830 and RUG11792 (see "Genome erosion and acquisition of new functions in *Endomicrobiellum*"). The transporter of RUG11792 clusters also with a homolog from endosymbionts of the family *Acutalibacteraceae* (*Oscillospirales*) (53). All these lineages of endosymbionts have been localized in flagellates of the genus *Trichonympha*, suggesting that the genes encoding NTTs may be frequently transferred among the endosymbionts of termite gut flagellates. By contrast, the NTTs of the putatively endosymbiotic *Opitutales*, which comprise the endonuclear *Candidatus* Nucleococcus from a termite gut flagellate (17) and several ciliate endosymbionts (80), are embedded among lineages from other environments, suggesting an ancestral presence in *Opitutales* (Fig. 6).

A hexuronate pathway occurs in the endosymbiotic genera *Endomicrobiellum*, *Ca*. Ancillula, and *Azobacteroides* (19, 21, 78) but also in other members of the family *Azobacteroidaceae*. Although *Endomicrobiaceae* apparently acquired the pathway from a clade of *Lachnospiraceae* associated with termites and ruminants, “*Ancillulaceae*” acquired it from *Lactobacillaceae* (Fig. S16). The origin of the hexuronate pathway in several putatively free-living *Endomicrobiaceae* differs from that in the genus *Endomicrobiellum* (Fig. S16). The capacity of these endosymbionts to degrade hexuronates may represent a convergent adaptation to their intracellular niche, because the hydrolysis of hemicelluloses in the digestive vacuoles of their flagellate hosts should yield large amounts of hexuronates (81).

The loss of pathways for the biosynthesis of amino acids in the genus *Endomicrobiellum* was compensated by the acquisition of several amino acid transporters absent from the ancestral, free-living lineages (Fig. 4). In the case of AroP, the homolog from *Endomicrobiellum* originates among homologs of “*Adiutricaceae*” (Fig. S17), whereas those of other *Endomicrobiaceae* cluster with homologs from putative endosymbionts in the order UBA3830. Again, the respective lineages co-colonize the same flagellates (*Trichonympha* spp.; [17]), providing further evidence that the convergent evolution of endosymbionts of termite gut flagellates is driven by horizontal gene transfer.

### Conclusions

Our comparative genomic analysis of *Endomicrobiaceae* provided new insights into the evolutionary processes underlying symbiogenesis in intracellular symbionts of termite gut flagellates. The transition from a free-living to an intracellular lifestyle not only restricts gene flow but also affects the ability to take up substrates and other nutrients from the environment. Members of the genus *Endomicrobiellum* possess functions that either represent adaptations to their intracellular niche or serve to compensate for gene losses during ongoing genome erosion. Some functions represent predispositions to an intracellular lifestyle that were present already in the common ancestor of *Endomicrobiaceae*, whereas others were apparently acquired by horizontal gene transfer. The acquisition of similar traits also by other established or putative flagellate symbionts underscores the importance of horizontal gene transfer from other gut microbiota in the convergent evolution of endosymbiotic lineages. The lack of biosynthetic functions common to all members of the genus *Endomicrobiellum* suggests that their original role in the symbiosis became compromised during the progressive genome reduction. It is possible that certain lineages now just represent a metabolic burden to their host. The acquisition of an NTT may indicate the transition from a mutualistic to a parasitic relationship, but it may also be the last straw to ensure survival for a beneficial endosymbiont whose energy metabolism became compromised owing to genome erosion. It is likely that dysfunctional members of *Endomicrobiellum* will be eventually replaced by secondary symbionts that co-occur in the same flagellates, as shown for other primary endosymbionts of eukaryotic hosts that have experienced severe gene losses (1, 82).

### Taxonomy

Most members of the family *Endomicrobiaceae* belong to genus-level lineages that are either unclassified or require reclassification. The presence of both high-quality genomes and 16S rRNA gene sequences for most lineages allow the proposal of new taxa under the Code of Nomenclature of Prokaryotes Described from Sequence Data (SeqCode) (83). The new names and new combinations, together with the designated type material, are listed in Table 1. The authors of previously proposed *Candidatus* names were assigned as descriptors of the corresponding new taxa. The full protologs including etymology, description, and other information are given in the supplemental material (Text S1).

**TABLE 1:** New genera in *Endomicrobiaceae* proposed under SeqCode, their designated type species, and other species assigned to the respective genus^*a*^

Genus	Type species	Other species in the genus
*Endomicrobium* (25)	*Endomicrobium proavitum* (25)	*Endomicrobium embiratermitis* sp. nov., *E. labiotermitis* sp. nov., *E. macrotermitis* sp. nov., *E. neocapritermitis* sp. nov., *E. procryptotermitis* sp. nov.
*Endomicrobiellum* gen. nov.	*Endomicrobiellum trichonymphae* sp. nov.	*Endomicrobiellum africanum* sp. nov., *Em. agilis* sp. nov., *Em. basalitermitum* sp. nov., *Em. calcaritermitis* sp. nov., *Em. calonymphae* sp. nov., *Em. cryptotermitis* sp. nov., *Em. cubanum* sp. nov., *Em. dinenymphae* sp. nov., *Em. glyptotermitis* sp. nov., *Em. guadaloupense* sp. nov., *Em. incisitermitis* sp. nov., *Em. mastotermitis* sp. nov., *Em. meruensis* sp. nov., *Em. neotermitis* sp. nov., *Em. porotermitis* sp. nov., *Em. pyrsonymphae* sp. nov., *Em. roisinitermitis* sp. nov., *Em. siamense* sp. nov.
*Ectomicrobium* gen. nov.	*Ectomicrobium neotermitis* sp. nov.	
*Parendomicrobium* gen. nov.	*Parendomicrobium reticulitermitis* sp. nov.	*Parendomicrobium porotermitis* sp. nov.
*Proendomicrobium* gen. nov.	*Proendomicrobium guianensium* sp. nov.	
*Ruminimicrobium* gen. nov.	*Ruminimicrobium bovinum* sp. nov.	
*Ruminimicrobiellum* gen. nov.	*Ruminimicrobiellum bubulum* sp. nov.	*Ruminimicrobiellum caprinum* sp. nov., *R. ovillum* sp. nov., *R. tauri* sp. nov.
*Praeruminimicrobium* gen. nov.	*Praeruminimicrobium purgamenti* sp. nov.	
*Proruminimicrobium* gen. nov.	*Proruminimicrobium quisquiliarum* sp. nov.	

^*a*^ The nomenclatural types (genomes) of all new species and their full protologs can be found in the supplemental material (Text S1).

## MATERIALS AND METHODS

### Metagenome sequencing

Termite colonies collected in the ﬁeld from various locations were sampled within a week of collection. Samples from termite colonies maintained in other laboratories were processed within a few days after arrival. Termite identification was confirmed by sequence analysis of the cytochrome oxidase II genes (84); sequences that were not represented in public databases have been submitted to NCBI GenBank. Details of each sample can be found in Table S1.

Worker termites were dissected with fine-tipped forceps. Gut suspensions of 30–50 individuals were either extracted directly or stored in 2-mL polypropylene tubes containing RNAlater (Invitrogen) at –20°C. DNA was extracted with a bead-beating protocol using the NucleoSpin Soil kit (Macherey-Nagel) as described previously (85). The extracted DNA was quantified with a Qubit fluorometer (Invitrogen), checked for purity with a NanoDrop ND-1000 spectrophotometer (PeqLab), and stored at –20°C.

Metagenomic libraries were sequenced using an Illumina NovaSeq 6000 platform in paired-end mode (2 × 150 bp) (Eurofins, Germany). Sequencing depth was 50 million read pairs for higher termites and 100 million read pairs for lower termites, accounting for a higher proportion of eukaryotic DNA contributed by their gut flagellates. The metagenomic data sets were submitted to the NCBI Sequence Read Archive (SRA) under the BioProject PRJNA732531 (for accession numbers, see Table S1).

### Sequence processing and genome recovery

Each of the 48 metagenomes was processed independently. First, we used the illumina-utils library v1.4.1 (86) to remove noise from raw reads with the program iu-filter-quality-minoche (87). Then, we identified host sequences by mapping our reads with Bowtie 2 v2.2.5 (88) against a database composed of the three termite genomes available at the time of the analysis: *Zootermopsis nevadensis* (89), *Cryptotermes secundus* (90), and *Macrotermes natalensis* (91). Subsequently, we removed the host reads by retaining only the unmapped reads using SAMtools v1.7 (92). Then, we assembled the reads of each metagenomic sample with MEGAHIT v1.2.8 (93) using a minimum contig length of 1,000 bp and a list of various *k*-mer sizes (21, 31, 41, 51, 61, 71, 81, 91, 99, 109, and 119). The coverage profile of each contig was computed with the bwa-mem algorithm from BWA v0.7.17 (94). These abundance scores combined with normalized tetranucleotide frequency score were used to bin the assembled reads with MetaBAT v2.14 (95).

The quality of the resulting MAGs was evaluated using CheckM v1.1.1 (96). The MAGs were classified using the Genome Taxonomy Database (GTDB) release 214 (97, 98) and the associated GTDB-Tk v2.3.0 (99, 100). The MAGs have been deposited at the NCBI Assembly Database under the accessions JAIRJQ000000000–JAITXW000000000. Detailed information on each MAG is listed in Table S2.

### Phylogenomic analysis

To infer phylogenetic relationships between the genomes, we collected a set of *Endomicrobiaceae* genomes and used *Endomicrobiales* genomes as outgroup. After checking the quality of all genomes with CheckM, we used GTDB-Tk to extract the concatenated and filtered alignment of 120 bacterial single-copy marker proteins (99). A maximum-likelihood tree was inferred with IQ-TREE v1.6.11 (101) employing the best-fitting model determined by ModelFinder based on the Bayesian information criterion (102). Branch support was assessed using ultrafast bootstrap approximation (UFBoot) (103, 104) and Shimodaira–Hasegawa approximate likelihood-ratio test (SH-aLRT) (105, 106).

### 16S rRNA gene phylogeny

A curated alignment of 16S rRNA genes of *Endomicrobiaceae* (11) was expanded with all 16S rRNA gene sequences of *Endomicrobiaceae* (>400 bp) extracted from the MAGs and termite gut metagenomes using Barrnap v0.9 (107). After manual curation, the alignment was exported and used to infer a maximum-likelihood tree using IQ-TREE with the best-fit evolutionary model suggested by ModelFinder; branch support was assessed with SH-aLRT and UFBoot analysis.

### Functional annotation

Open reading frames were predicted and annotated for the *Endomicrobia* MAGs and all previously published genomes and genomic scaffolds using Prokka version 1.14.5 (108), with the following settings: -kingdom Bacteria, -increment 1, -mincontiglen 200, -evalue 1e-06.

For functional annotation, the generated protein files were investigated by Hidden Markov Model (HMM) searching in HMMER version 3.3 using hmmsearch (109) and the HMMs from the Pfam database (releases 31.0, 33.1) (110) and the TIGRFAM database (release 15.0) (111). Transporters and hydrogenases were annotated using the Transporter Classification Database (TCDB) (112) and the hydrogenase database (HydDB) (113). The results were verified using BLASTp version 2.6.0 (114) against a custom database that consisted of the annotated *Endomicrobiaceae* genomes and the SWISS-PROT database (115), which provides manually annotated, non-redundant protein sequences of high quality.

Transposase genes and insertion sequences (IS) were predicted using Issaga (116; http://issaga.biotoul.fr/issaga_index.php). The clustered regularly interspaced palindromic repeats (CRISPR) and intergenic spacer sequences were identified using CRISPR recognition tool (117); the related *cas* genes in the genomes were identified by BLASTp searches against the NCBI non-redundant protein sequence database.

### Protein phylogenies

Amino acid sequences of genes were recovered from the *Endomicrobiaceae* MAGs and were expanded with SWISS-PROT entries. The deduced sequences were aligned with MAFFT version 7.427 and were used to calculate a first gene tree in IQTREE using the best-fitting model of evolution suggested by ModelFinder. Node support was assessed with UFBoot (103, 104) and SH-aLRT (105, 106). The alignment and resulting consensus tree were imported into ARB version 6.1 (118), where the alignment was curated manually and exported again to calculate a new tree using the same settings as above.

## Data Availability

The metagenomic data sets were submitted to the NCBI Sequence Read Archive (SRA) under the BioProject PRJNA732531 (for accession numbers, see Table S1). The MAGs have been deposited at the NCBI Assembly Database under the accessions JAIRJQ000000000–JAITXW000000000.

## SUPPLEMENTAL MATERIAL

Text S1 mbio.00826-24-s0001.pdf Protologs for new prokaryotic taxa. Etymology and description of the new taxa proposed under SeqCode.

Supplemental Figures mbio.00826-24-s0002.pdf Fig. S1–S17.

Fig. S15 mbio.00826-24-s0003.html Interactive HTML file.

Supplemental Tables mbio.00826-24-s0004.xlsx Tables S1–S7.

ASM does not own the copyrights to Supplemental Material that may be linked to, or accessed through, an article. The authors have granted ASM a non-exclusive, world-wide license to publish the Supplemental Material files. Please contact the corresponding author directly for reuse.
