# Supplementary material for: Genome reduction and horizontal gene transfer in the evolution of Endomicrobia—rise and fall of an intracellular symbiosis with termite gut flagellates
Source: mBio. 2024 May 14;15(6):e00826-24. doi: 10.1128/mbio.00826-24 (PMC11257099; doi:10.1128/mbio.00826-24)
Supplement: Text S1 — Protologs for new prokaryotic taxa. Etymology and description of the new taxa proposed under SeqCode. [file mbio.00826-24-s0001.pdf]

Supplementary Material – Text S1

Protologs for new prokaryotic taxa

Etymology and description of the new taxa proposed under SeqCode.

Endomicrobiaceae Zheng et al. 2018 emend.

Etymology: En.do.mi.cro.bi.a'ce.ae. N.L. neutr. n. *Endomicrobium*, type genus of the family; L. fem. pl. n. suff. -*aceae*, ending to denote a family; N.L. fem. pl. n. *Endomicrobiaceae*, the *Endomicrobium* family.

Description: Gram-negative cell envelope. Non motile. Obligately anaerobic and catalase negative. Heterotrophic, purely fermentative metabolism. Occur in the intestinal tracts of insects and ruminants. Members are free-living, attached to the surface of protists, or intracellular symbionts of protists.

Type genus: *Endomicrobium*

Endomicrobium Zheng et al. 2018 emend.

Etymology: En.do.mi.cro'bi.um. Gr. pref. *endo*-, within; N.L. neut. n. *microbium*, microbe; N.L. neut. n. *Endomicrobium*, a microbe that occurs within (another organism).

Description: The genus comprises isolates and metagenome-assembled genomes. Occur free-living in the hindgut of termites and cockroaches. The genus is defined by phylogenomic analysis as a monophyletic group that shows a relative evolutionary divergence (RED) similar to that of the neighboring genera.

Type species: *Endomicrobium proavitum*

Endomicrobium proavitum Zheng et al. 2018 emend.

Etymology: pro.a'vi.tum. L. neut. adj. *proavitum* (from L. masc. n. *proavus*, ancestor), belonging to the ancestors (of the intracellular symbionts in the genus *Endomicrobiellum*).

Description: The description remains the same as in Zheng et al. (2016), with the following amendments. The species includes all bacteria with more than 95% average nucleotide identity (ANI) to the type strain. The GC content of the type strain is 39.3% and the estimated genome size is 1.6 Mbp.

Type strain: Rsa215^{T} = DSM 29378^{T} = JCM 30189^{T}

Type genome: Rsa215^{TS} = GCF_001027545.1^{TS}; NR_156018.1 (16S rRNA gene)

Endomicrobium embiratermitis sp. nov. Mies and Brune

Etymology: em.bi.ra.ter'mi.tis. N.L. gen. n. *embiratermitis*, of *Embiratermes*, referring to the termite host genus.

Description: The species comprises only metagenome-assembled genomes. The species includes all bacteria with more than 95% average nucleotide identity (ANI) to the type genome. The GC content of the type genome is 39.6% and the estimated genome size is 2.0 Mbp.

Type genome: Emb289P3_bin85^{TS} = GCA_009781315.1^{TS}

Endomicrobium labiotermitis sp. nov. Mies and Brune

Etymology: la.bi.o.ter'mi.tis. N.L. gen. n. *labiotermitis*, of *Labiotermes*, referring to the termite host genus.

Description: The species comprises only metagenome-assembled genomes. The species includes all bacteria with more than 95% average nucleotide identity (ANI) to the type genome. The GC content of the type genome is 38.0% and the estimated genome size is 2.0 Mbp.

Type genome: Lab288P4_bin38^{TS} = GCA_009778965.1^{TS}

Endomicrobium macrotermitis sp. nov. Mies and Brune

Etymology: ma.cro.ter'mi.tis. N.L. gen. n. *macrotermitis*, of *Macrotermes*, referring to the termite host genus.

Description: The species comprises only metagenome-assembled genomes. The species includes all bacteria with more than 95% average nucleotide identity (ANI) to the type genome. The GC content of the type genome is 42.7% and the estimated genome size is 2.0 Mbp.

Type genome: Mx356_bin47^{TS} = GCA_031271405.1^{TS}

Endomicrobium neocapritermitis sp. nov. Mies and Brune

Etymology: ne.o.ca.pri.ter'mi.tis. N.L. gen. n. *neocapritermitis*, of *Neocapritermes*, referring to the termite host genus.

Description: The species comprises only metagenome-assembled genomes. The species includes all bacteria with more than 95% average nucleotide identity (ANI) to the type genome. The GC content of the type genome is 39.7% and the estimated genome size is 2.4 Mbp.

Type genome: Nt197P4_bin29^{TS} = GCA_009778325.1^{TS}

Endomicrobium procryptotermitis sp. nov. Mies and Brune

Etymology: pro.cryp.to.ter'mi.tis. N.L. gen. n. *procryptotermitis*, of *Procryptotermes*, referring to the termite host genus.

Description: The species comprises only metagenome-assembled genomes. The species includes all bacteria with more than 95% average nucleotide identity (ANI) to the type genome. The GC content of the type genome is 36.3% and the estimated genome size is 2.1 Mbp.

Type genome: Pcl387_bin121^{TS} = GCA_031279415.1^{TS}

Endomicrobiellum gen. nov. Mies and Brune

Etymology: En.do.mi.cro.bi.ell'um. Gr. pref. *endo-*, within; N.L. dim. neut. n. *microbiellum*, a small microbe; N.L. neut. n. *Endomicrobiellum*, a small microbe that occurs within (a host cell).

Description: A bacterial genus identified by genomic, single-cell amplified genomes and metagenome-assembled genome. All members of the genus are intracellular symbionts of termite gut flagellates. The genus is defined by phylogenomic analysis as a monophyletic group that shows a relative evolutionary divergence (RED) similar to that of the neighboring genera.

Type species: *Endomicrobiellum trichonymphae*

Endomicrobiellum trichonymphae sp. nov. Stingl et al.

Etymology: tri.cho.nym'phae. N.L. gen. n. *trichonymphae*, of *Trichonympha*, referring to the host flagellate.

Synonym: "*Candidatus* Endomicrobium trichonymphae" Stingl et al. 2005

Description: The species comprises only single-cell amplified genomes. Colonizes the cytoplasm of the cellulolytic protist *Trichonympha agilis* in the termite gut. Spindle-shaped cells (0.6 µm in length and 0.3 µm in diameter). The outer membrane forms tubular extensions into the cytoplasm of the host. Localized in the cytoplasm of flagellates of the genus *Trichonympha* using FISH with a specific oligonucleotide probe. The species includes all bacteria with more than 95% average nucleotide identity (ANI) to the type genome. The GC content of the type strain is 35.2% and the estimated genome size is 1.1Mbp.

Type genome: Ti2015^{TS} = GCA_002355835.1^{TS}; AP017459.1 (16S rRNA gene)

Additional genomes: GCA_000146025.1

Endomicrobiellum agilis sp. nov. Stephens et al.

Etymology: a'gi.lis. L. gen. n. *agilis*, of *Trichonympha agilis,* referring to the host species.

Synonym: "*Candidatus* Endomicrobium agilae" Stephens et al. 2022.

Description: The species comprises only metagenome-assembled genomes and single-cell amplified genomes. The species includes all bacteria with more than 95% average nucleotide identity (ANI) to the type genome. The GC content of the type genome is 36.0% and the estimated genome size is 1.3 Mbp.

Type genome: TA26^{TS} = GCA_020328155.1^{TS}

Additional genomes: GCA_020328115.1; GCA_031286885.1

Endomicrobiellum africanum sp. nov. Mies and Brune

Etymology: a.fri.ca'.num. N.L. neut. adj. *africanum*, of Africa, African, referring to the geographic origin of the host genus.

Description: The species comprises only metagenome-assembled genomes. The species includes all bacteria with more than 95% average nucleotide identity (ANI) to the type genome. The GC content of the type genome is 31.2% and the estimated genome size is 1.1 Mbp.

Type genome: Nm470_bin63^{TS} = GCA_031281995.1^{TS}; OR396879 (16S rRNA gene)

Additional genomes: GCA_031266185.1

Endomicrobiellum basalitermitum sp. nov. Mies and Brune

Etymology: ba.sa.li.ter'mi.tum. L. adj. *basalis*, basal; L. masc. n. *termes*, a woodworm, termite; N.L. gen. pl. masc. n. *basalitermitum*, of lower (basal) termites.

Description: The species comprises only metagenome-assembled genomes. The species includes all bacteria with more than 95% average nucleotide identity (ANI) to the type genome. The GC content of the type genome is 30.8% and the estimated genome size is 0.8 Mbp.

Type genome: Nm470_bin135^{TS} = GCA_031282525.1^{TS}

Endomicrobiellum devescovinae sp. nov. Mies and Brune

Etymology: de.ves.co.vi'nae. N.L. gen. n. *devescovinae*, of *Devescovina*, referring to the flagellate host.

Description: The species comprises only metagenome-assembled genomes. The species includes all bacteria with more than 95% average nucleotide identity (ANI) to the type genome. The GC content of the type genome is 34.1% and the estimated genome size is 1.2 Mbp.

Type genome: Ct408_bin22^{TS} = GCA_031255495.1^{TS}; OR396872 (16S rRNA gene)

Additional genomes: GCA_031267795.1; GCA_031274155.1; GCA_031279375.1; GCA_031284395.1

Endomicrobiellum calonymphae sp. nov. Mies and Brune

Etymology: ca.lo.nym'phae. N.L. gen. fem. n. *calonymphae*, of *Calonympha*, referring to the host flagellate.

Description: The species comprises only metagenome-assembled genomes. The species includes all bacteria with more than 95% average nucleotide identity (ANI) to the type genome. The GC content of the type genome is 32.4% and the estimated genome size is 0.9 Mbp.

Type genome: Nc350_bin26^{TS} = GCA_031269945.1^{TS}

Endomicrobiellum cryptotermitis sp. nov. Mies and Brune

Etymology: cryp.to.ter'mi.tis. N.L. gen. n., *cryptotermitis*, of *Cryptotermes*, referring to the termite host genus.

Description: The species comprises only metagenome-assembled genomes. The species includes all bacteria with more than 95% average nucleotide identity (ANI) to the type genome. The GC content of the type genome is 31.1% and the estimated genome size is 1.2 Mbp.

Type genome: Cd354_bin52^{TS} = GCA_031257835.1^{TS}

Endomicrobiellum cubanum sp. nov. Mies and Brune

Etymology: cu.ba'num. N.L. neut. adj. *cubanum*, of Cuba, Cuban, referring to the geographic origin of the host genus.

Description: The species comprises only metagenome-assembled genomes. The species includes all bacteria with more than 95% average nucleotide identity (ANI) to the type genome. The GC content of the type genome is 31.1% and the estimated genome size is 1.4 Mbp.

Type genome: Nc350_bin45^{TS} = GCA_031269735.1^{TS}; OR396877 (16S rRNA gene)

Additional genomes: GCA_031278955.1

Endomicrobiellum dinenymphae sp. nov. Stephens et al.

Etymology: di.ne.nym'phae. N.L. gen. n. *dinenymphae*, of *Dinenympha,* referring to the host flagellate.

Synonym: "*Candidatus* Endomicrobium dinenymphae" Stephens et al. 2022

Description: The species comprises only single-cell amplified genomes. Colonizes the cytoplasm of *Dinenympha*. The species includes all bacteria with more than 95% average nucleotide identity (ANI) to the type genome. The GC content of the type strain is 36.0% and the estimated genome size is 1.1Mbp.

Type genome: DS12^{TS} = GCA_020328135.1^{TS}

Endomicrobiellum glyptotermitis sp. nov. Mies and Brune

Etymology: glyp.to.ter'mi.tis. N.L. gen. n. *glyptotermitis*, of *Glyptotermes*, referring to the termite host genus.

Description: The species comprises only metagenome-assembled genomes. The species includes all bacteria with more than 95% average nucleotide identity (ANI) to the type genome. The GC content of the type genome is 33.5% and the estimated genome size is 0.8 Mbp.

Type genome: Gsp477_bin5^{TS} = GCA_031267715.1^{TS}

Endomicrobiellum guadaloupense sp. nov. Mies and Brune

Etymology: gu.a.da.lu'pi.ae. N.L. gen. neutr. adj. *guadaloupense*, of Guadaloupe, referring to the geographic origin of the host genus.

Description: The species comprises only metagenome-assembled genomes. The species includes all bacteria with more than 95% average nucleotide identity (ANI) to the type genome. The GC content of the type genome is 32.5% and the estimated genome size is 1.3 Mbp.

Type genome: Pcl387_bin170^{TS} = GCA_031278895.1^{TS}

Endomicrobiellum incisitermitis sp. nov. Mies and Brune

Etymology: in.ci.si.ter'mi.tis. N.L. gen. n. *incisitermitis*, of *Incisitermes*, referring to the termite host genus.

Description: The species comprises only metagenome-assembled genomes. The species includes all bacteria with more than 95% average nucleotide identity (ANI) to the type genome. The GC content of the type genome is 31.2% and the estimated genome size is 1.3 Mbp.

Type genome: Iy174_bin31^{TS} = GCA_031275795.1^{TS}

Endomicrobiellum mastotermitis sp. nov. Mies and Brune

Etymology: mas.to.ter'mi.tis. N.L. gen. n. *mastotermitis*, of *Mastotermes*, referring to the termite host genus.

Description: The species comprises only metagenome-assembled genomes. The species includes all bacteria with more than 95% average nucleotide identity (ANI) to the type genome. The GC content of the type genome is 31.0% and the estimated genome size is 0.9 Mbp.

Type genome: Md513_bin31^{TS} = GCA_031272445.1^{TS}; OR396874 (16S rRNA gene)

Endomicrobiellum meruensis sp. nov. Mies and Brune

Etymology: me.ru.en'sis. N.L. gen. n. *meruensis*, of *Neotermes meruensis*, referring to the host species.

Description: The species comprises only metagenome-assembled genomes. The species includes all bacteria with more than 95% average nucleotide identity (ANI) to the type genome. The GC content of the type genome is 33.8% and the estimated genome size is 1.3 Mbp.

Type genome: Nm470_bin124^{TS} = GCA_031282625.1^{TS}

Additional genomes: GCA_031265775.1

Endomicrobiellum neotermitis sp. nov. Mies and Brune

Etymology: ne.o.ter'mi.tis. N.L. gen. n. *neotermitis*, of *Neotermes*, referring to the termite host genus.

Description: The species comprises only metagenome-assembled genomes. The species includes all bacteria with more than 95% average nucleotide identity (ANI) to the type genome. The GC content of the type genome is 29.0% and the estimated genome size is 0.6 Mbp.

Type genome: Nm470_bin18^{TS} = GCA_031282355.1^{TS}; OR396870 (16S rRNA gene)

Endomicrobiellum porotermitis sp. nov. Mies and Brune

Etymology: po.ro.ter'mi.tis. N.L. gen. n. *porotermitis*, of *Porotermes*, referring to the termite host genus.

Description: The species comprises only metagenome-assembled genomes. The species includes all bacteria with more than 95% average nucleotide identity (ANI) to the type genome. The GC content of the type genome is 37.2% and the estimated genome size is 1.1 Mbp.

Type genome: Pq454_bin6^{TS} = GCA_031289055.1^{TS}; OR396880 (16S rRNA gene)

Additional genomes: GCA_031269295.1

Endomicrobiellum pyrsonymphae sp. nov. Stingl et al.

Etymology: pyr.so.nym'phae. N.L. gen. n. *pyrsonymphae*, of *Pyrsonympha*, referring to the host flagellate.

Synonym: "*Candidatus* Endomicrobium pyrsonymphae" Stingl et al. 2005.

Description: The species comprises only single-cell amplified genomes. Colonizes the cytoplasm of *Pyrsonympha vertens*. Localized in the cytoplasm of *Pyrsonympha vertens* using FISH with a specific oligonucleotide probe. The species includes all bacteria with more than 95% average nucleotide identity (ANI) to the type genome. The GC content of the type strain is 35.3% and the estimated genome size is 1.3Mbp.

Type genome: PV1^{TS} = GCA_020328045.1^{TS}

Additional genomes: GCA_020328025.1

Endomicrobiellum roisinitermitis sp. nov. Mies and Brune

Etymology: roi.si.ni.ter'mi.tis. N.L. gen. n. *roisinitermitis*, of *Roisinitermes*, referring to the termite host genus.

Description: The species comprises only metagenome-assembled genomes. The species includes all bacteria with more than 95% average nucleotide identity (ANI) to the type genome. The GC content of the type genome is 31.3% and the estimated genome size is 1.3 Mbp.

Type genome: Roe453_bin8^{TS} = GCA_031287855.1^{TS}; OR396878 (16S rRNA gene)

Endomicrobiellum siamense sp. nov. Mies and Brune

Etymology: si.a.men'se. N.L. neut. adj. *siamense*, of Siam, Thai, referring to the geographic origin of the host genus.

Description: The species comprises only metagenome-assembled genomes. The species includes all bacteria with more than 95% average nucleotide identity (ANI) to the type genome. The GC content of the type genome is 30.8% and the estimated genome size is 1.3 Mbp.

Type genome: Gsp477_bin175^{TS} = GCA_031268145.1^{TS}; OR396875 (16S rRNA gene)

Parendomicrobium gen. nov. Mies and Brune

Etymology: Par.en.do.mi.cro'bi.um. L. adj. *par*, equal, like; N.L. neut. n. *Endomicrobium*, a bacterial genus; N.L. neut. n. *Parendomicrobium*, a bacterial genus like *Endomicrobium*.

Description: A bacterial genus defined by metagenome-assembled genomes. The genus is defined by phylogenomic analysis as a monophyletic group that shows a relative evolutionary divergence (RED) similar to that of the neighboring genera.

Type species: *Parendomicrobium reticulitermitis*

Parendomicrobium reticulitermitis sp. nov. Mies and Brune

Etymology: re.ti.cu.li.ter'mi.tis. N.L. gen. n. *reticulitermitis*, of *Reticulitermes*, referring to the termite host genus.

Description: The species comprises only metagenome-assembled genomes. The species includes all bacteria with more than 95% average nucleotide identity (ANI) to the type genome. The GC content of the type genome is 37.7% and the estimated genome size is 2.3 Mbp.

Type genome: Rs511_bin47^{TS} = GCA_031287095.1^{TS}

Parendomicrobium porotermitis sp. nov. Mies and Brune

Etymology: po.ro.ter'mi.tis. N.L. gen. n. *prorotermitis*, of *Porotermes*, referring to the termite host genus.

Description: The species comprises only metagenome-assembled genomes. The species includes all bacteria with more than 95% average nucleotide identity (ANI) to the type genome. The species includes all bacteria with more than 95% average nucleotide identity (ANI) to the type genome. The GC content of the type genome is 35.3% and the estimated genome size is 1.4 Mbp.

Type genome: Po218_bin99^{TS} = GCA_031289555.1^{TS}

Ectomicrobium gen. nov. Mies and Brune

Etymology: Ec.to.mi.cro'bi.um. Gr. adv. *ektos*, outside, external; N.L. neut. n. *microbium*, microbe; N.L. neut. n. *Ectomicrobium*, a microbe that occurs on the outside (of another organism).

Description: A bacterial genus defined by metagenome-assembled genomes. The genus is defined by phylogenomic analysis as a monophyletic group that shows a relative evolutionary divergence (RED) similar to that of the neighboring genera.

Type species: *Ectomicrobium neotermitis*

Ectomicrobium neotermitis sp. nov. Mies and Brune

Etymology: ne.o.ter'mi.tis. N.L. gen. n. *neotermitis*, of *Neotermes*, referring to the termite host genus.

Description: The species comprises only metagenome-assembled genomes. The species includes all bacteria with more than 95% average nucleotide identity (ANI) to the type genome. The GC content of the type genome is 41.2% and the estimated genome size is 1.4 Mbp.

Type genome: Ncb351_bin78^{TS} = GCA_031283745.1^{TS}; OR396873 (16S rRNA gene)

Proendomicrobium gen. nov. Mies and Brune

Etymology: Pro.en.do.mi.cro'bi.um. L. pref. *pro*, (temporally) prior, fore-; N.L. neut. n. *Endomicrobium*, a bacterial genus; N.L. neut. n. *Proendomicrobium*, a bacterial genus ancestral to *Endomicrobium*.

Description: A bacterial genus defined by metagenome-assembled genomes. The genus is defined by phylogenomic analysis as a monophyletic group that shows a relative evolutionary divergence (RED) similar to that of the neighboring genera.

Type species: *Proendomicrobium guianensium*

Proendomicrobium guianensium sp. nov. Mies and Brune

Etymology: gu.i.a.nen'si.um. N.L. pl. fem. n. *Guianae*, the Guianas, a region in north-eastern South America, L. gen. pl. suff. *-ensium*, of or from a place; N.L. gen. pl. adj. *guianensium*, of the Guianas, referring to the geographic origin of the host genus.

Description: The species comprises only metagenome-assembled genomes. The species includes all bacteria with more than 95% average nucleotide identity (ANI) to the type genome. The GC content of the type genome is 41.2% and the estimated genome size is 1.4Mbp.

Type genome: Emb289P1 bin39^{TS} = GCA_009785775.1^{TS}

Ruminimicrobium gen. nov. Mies and Brune

Etymology: Ru.mi.ni.mi.cro'bi.um. L. neut. n. *rumen* (gen. *ruminis*), the rumen; N.L. neut. n. *microbium*, microbe; N.L. neut. n. *Ruminimicrobium*, a microbe that occurs in the rumen.

Description: A bacterial genus defined by metagenome-assembled genomes. The genus is defined by phylogenomic analysis as a monophyletic group that shows a relative evolutionary divergence (RED) similar to that of the neighboring genera.

Type species: *Ruminimicrobium bovinum*

Ruminimicrobium bovinum sp. nov. Mies and Brune

Etymology: bo.vi'num. L. neut. adj. *bovinum*, of or belonging to cattle, referring to the isolation source

Description: The species comprises only metagenome-assembled genomes. The species includes all bacteria with more than 95% average nucleotide identity (ANI) to the type genome. The GC content of the type genome is 31.0% and the estimated genome size is 1.9 Mbp.

Type genome: RUG240^{TS} = GCA_900316875.1^{TS}

Additional genomes: GCA_902760015.1

Ruminimicrobiellum gen. nov. Mies and Brune

Etymology: Ru.mi.ni.mi.cro'bi.ell.um. L. neut. n. *rumen* (gen. *ruminis*), the rumen; N.L. dim. neut. n. *microbiellum*, a small microbe; N.L. neut. n. *Ruminimicrobiellum*, a small microbe that occurs in the rumen.

Description: A bacterial genus defined by metagenome-assembled genomes. The genus is defined by phylogenomic analysis as a monophyletic group that shows a relative evolutionary divergence (RED) similar to that of the neighboring genera.

Type species: *Ruminimicrobiellum bubulum*

Ruminimicrobiellum bubulum sp. nov. Mies and Brune

Etymology: bu'bu.lum. L. neut. adj. *bubulum*, of or pertaining to cattle, referring to the isolation source.

Description: The species comprises only metagenome-assembled genomes. The species includes all bacteria with more than 95% average nucleotide identity (ANI) to the type genome. The GC content of the type genome is 31.8% and the estimated genome size is 1.9 Mbp.

Type genome: RGIG9669^{TS} = GCA_017651625.1^{TS}

Ruminimicrobiellum ovillum sp. nov. Mies and Brune

Etymology: o.vil'lum. L. neut. adj. *ovillum*, of or belonging to sheep, referring to the isolation source.

Description: The species comprises only metagenome-assembled genomes. The species includes all bacteria with more than 95% average nucleotide identity (ANI) to the type genome. The GC content of the type genome is 32.3% and the estimated genome size is 1.8 Mbp.

Type genome: UBA7019^{TS} = GCA_002448285.1^{TS}

Additional genomes: GCA_002389965.1; GCA_002369435.1; GCA_002392975.1

Ruminimicrobiellum caprinum sp. nov. Mies and Brune

Etymology: ca.pri'num. L. neut. adj. *caprinum*, of or belonging to goats, referring to the isolation source.

Description: The species comprises only metagenome-assembled genomes. The species includes all bacteria with more than 95% average nucleotide identity (ANI) to the type genome. The GC content of the type genome is 33.0% and the estimated genome size is 1.7 Mbp.

Type genome: RGIG1474^{TS} = GCA_017413305.1^{TS}

Ruminimicrobiellum tauri sp. nov. Mies and Brune

Etymology: tau'ri. L. masc. gen. n. *tauri*, of a steer, referring to the isolation source.

Description: The species comprises only metagenome-assembled genomes. The species includes all bacteria with more than 95% average nucleotide identity (ANI) to the type genome. The GC content of the type genome is 29.3% and the estimated genome size is 1.5 Mbp.

Type genome: RUG13817^{TS} = GCA_902792865.1^{TS}

Praeruminimicrobium gen. nov. Mies and Brune

Etymology: Prae.ru.mi.ni.mi.cro'bi.um. L. pref. *prae-*, before; N.L. neut. n. *Ruminimicrobium*, a microbe that occurs in the rumen; N.L. neut. n. *Praeruminimicrobium*, a bacterial genus ancestral to *Ruminimicrobium*.

Description: A bacterial genus defined by metagenome-assembled genomes. The genus is defined by phylogenomic analysis as a monophyletic group that shows a relative evolutionary divergence (RED) similar to that of the neighboring genera.

Type species: *Praeruminimicrobium purgamenti*

Praeruminimicrobium purgamenti sp. nov. Mies and Brune

Etymology: pur.ga.men'ti. L. gen. n. *purgamenti*, from waste, referring to the isolation source.

Description: The species comprises only metagenome-assembled genomes. The species includes all bacteria with more than 95% average nucleotide identity (ANI) to the type genome. The GC content of the type genome is 34.6% and the estimated genome size is 1.9 Mbp.

Type genome: $SV1^{TS}$ = $GCA\_018433585.1^{TS}$

Proruminimicrobium gen. nov. Mies and Brune

Etymology: Pro.ru.mi.ni.mi.cro'bi.um. L. pref. *pro*, (temporally) prior, fore-; N.L. neut. n. *Ruminimicrobium*, a microbe that occurs in the rumen; N.L. neut. n. *Proruminimicrobium*, a bacterial genus ancestral to *Ruminimicrobium*.

Description: A bacterial genus defined by metagenome-assembled genomes. The genus is defined by phylogenomic analysis as a monophyletic group that shows a relative evolutionary divergence (RED) similar to that of the neighboring genera.

Type genome: *Proruminimicrobium quisquiliarum*

Proruminimicrobium quisquiliarum sp. nov. Mies and Brune

Etymology: quis.qui.li.a'rum. L. gen. pl. n. *quisquiliarum*, from waste, referring to the isolation source.

Description: The species comprises only metagenome-assembled genomes. The species includes all bacteria with more than 95% average nucleotide identity (ANI) to the type genome. The GC content of the type genome is 35.1% and the estimated genome size is 2.0 Mbp.

Type genome: $SV1^{TS}$ = $GCA\_018433245.1^{TS}$
