# Supplementary material for: Genome reduction and horizontal gene transfer in the evolution of Endomicrobia—rise and fall of an intracellular symbiosis with termite gut flagellates
Source: mBio. 2024 May 14;15(6):e00826-24. doi: 10.1128/mbio.00826-24 (PMC11257099; doi:10.1128/mbio.00826-24)
Supplement: Supplemental Figures — Fig. S1–S17. [file mbio.00826-24-s0002.pdf]

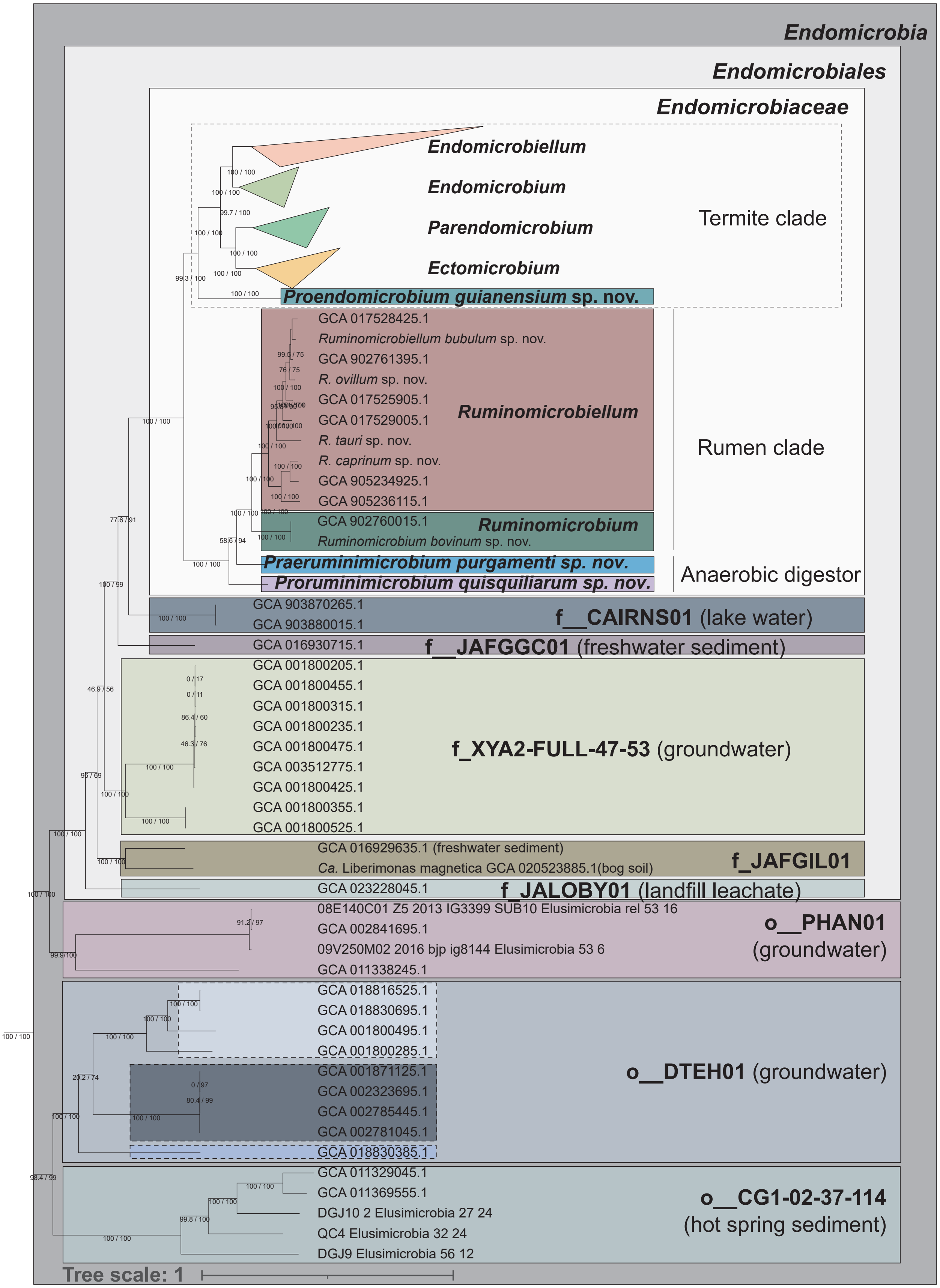

Figure S1: Phylogenomic tree of the class *Endomicrobia*. The maximum-likelihood tree was inferred from the concatenated amino acid alignment of 120 protein-coding genes generated by GTDB-Tk using the LG+F+I+G4 model of evolution. Values on internal nodes indicate SH-aLRT/UFBoot support. Genomes without Genbank accession number were published in (63) and downloaded from ggkbase.

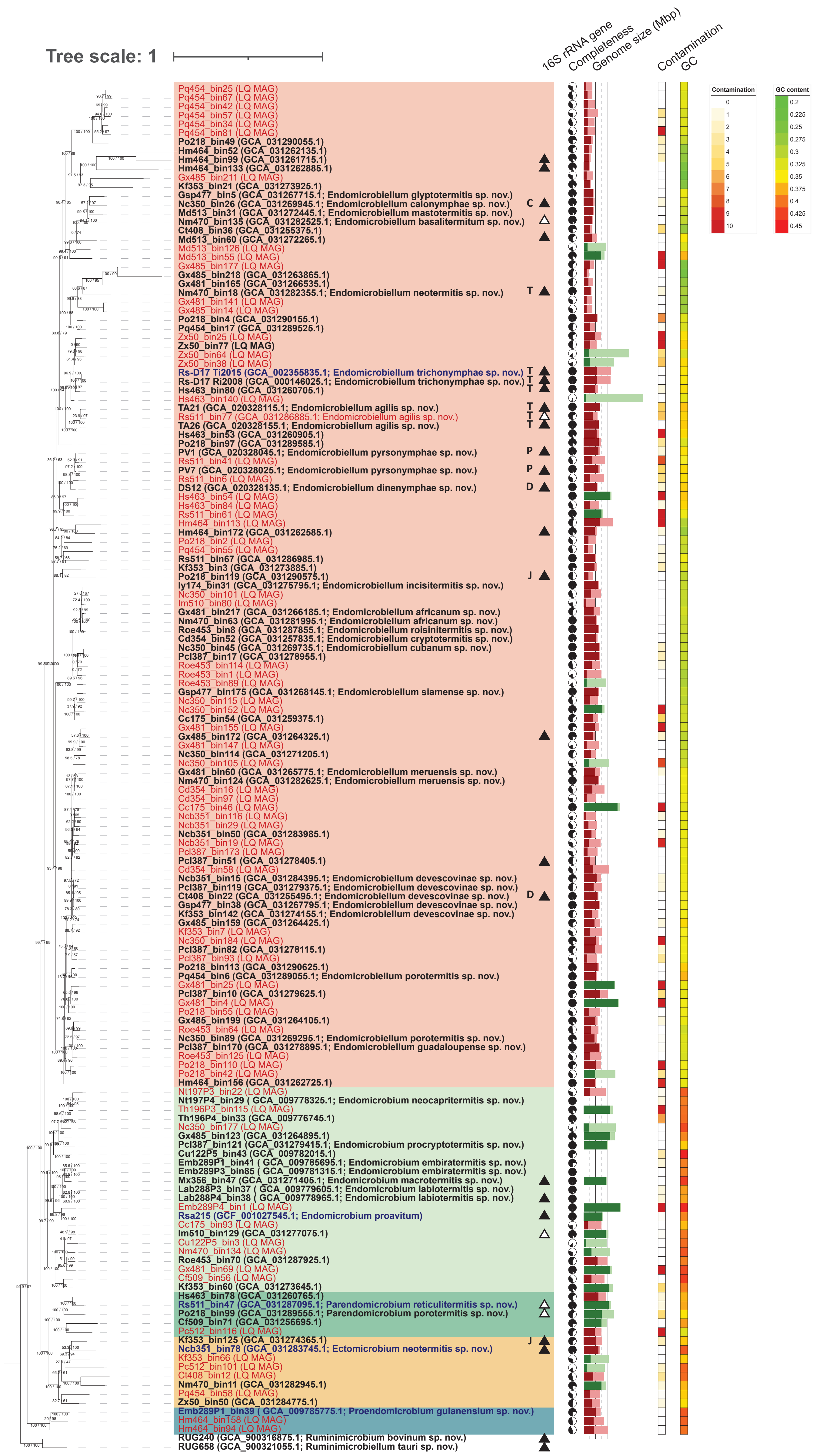

Figure S2: Phylogenomic tree of *Endomicrobiaceae*, including the accession numbers of all MAGs in Figure 3. Additional, low-quality MAGs (< 50% completeness, < 10% contamination) from the corresponding metagenomes are indicated in red. The maximum-likelihood tree was inferred from the concatenated amino acid alignment of 120 protein-coding genes generated by GTDB-Tk using the LG+F+I+G4 model of evolution. Values on internal nodes indicate SH-aLRT/UFBoot support. A filled triangle marks genomes with 16S rRNA genes, an open triangle indicates a representation among homologs from the corresponding metagenomes (see Figure S3). Established associations with termite gut flagellates are indicated with letters (J, *Joenia*; D, *Dinenympha*; P, *Pyrsonympha*; T, *Trichonympha*; C, *Calonympha*). Genome size was estimated from assembly size and completeness, as determined by CheckM (indicated by shaded and filled bars).

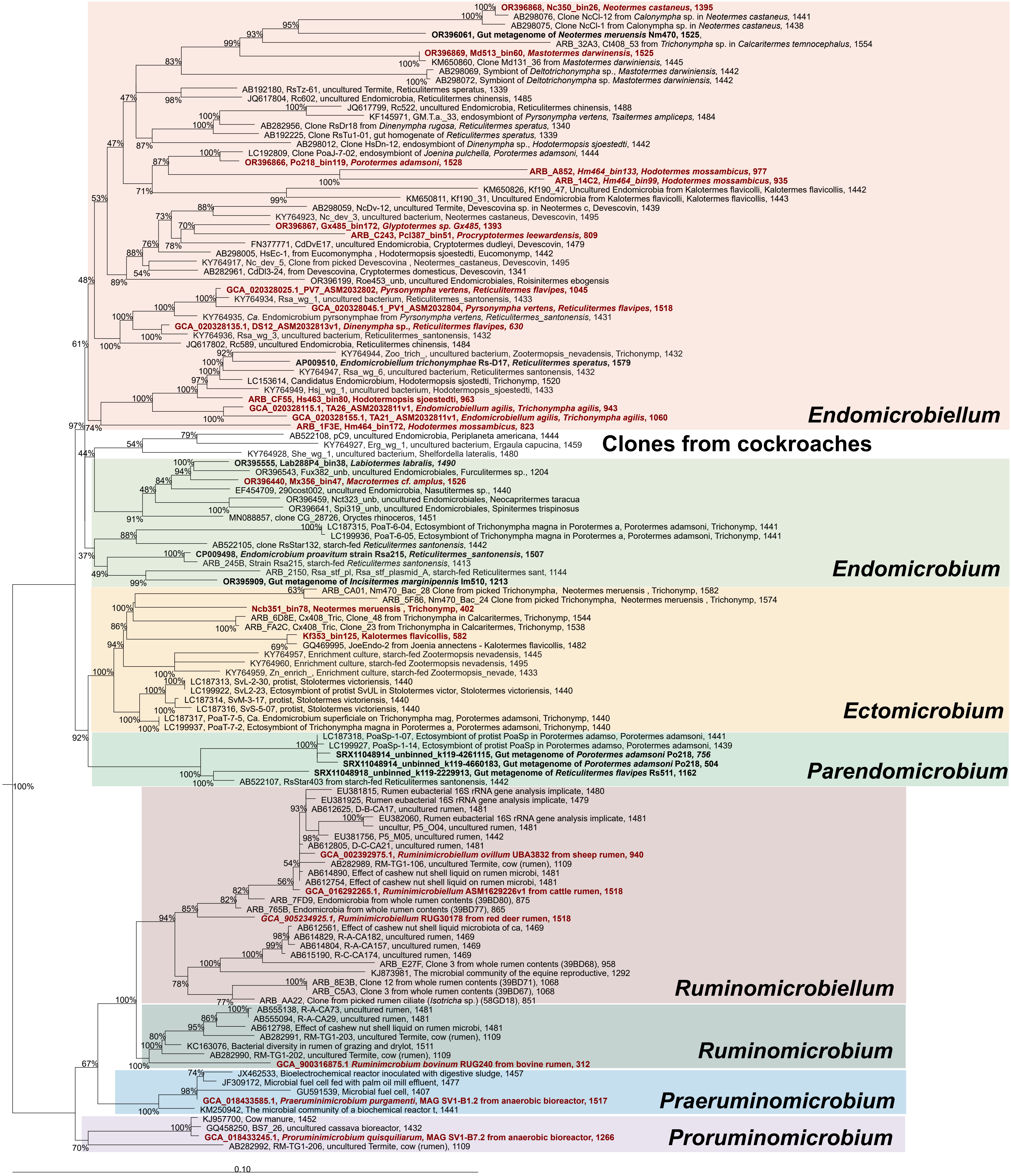

Figure S3: Phylogenetic tree of *Endomicrobiaceae*, based on a curated alignment of the 16S rRNA gene (1,464 positions) inferred under a GTR+F +R4 model of evolution. The tree was rooted with *Endomicrobiales* and illustrates the consensus topology of an SH-aLRT and a UFBoot analysis (1,000 replicates each). Homologs from MAGs (red) and metagenomes (black) are indicated in boldface. Filled circles illustrate a consensus support threshold ≥ 90%, empty circles a consensus support threshold ≥ 70%. The scale bar indicates 0.1 nucleotide substitutions per site.

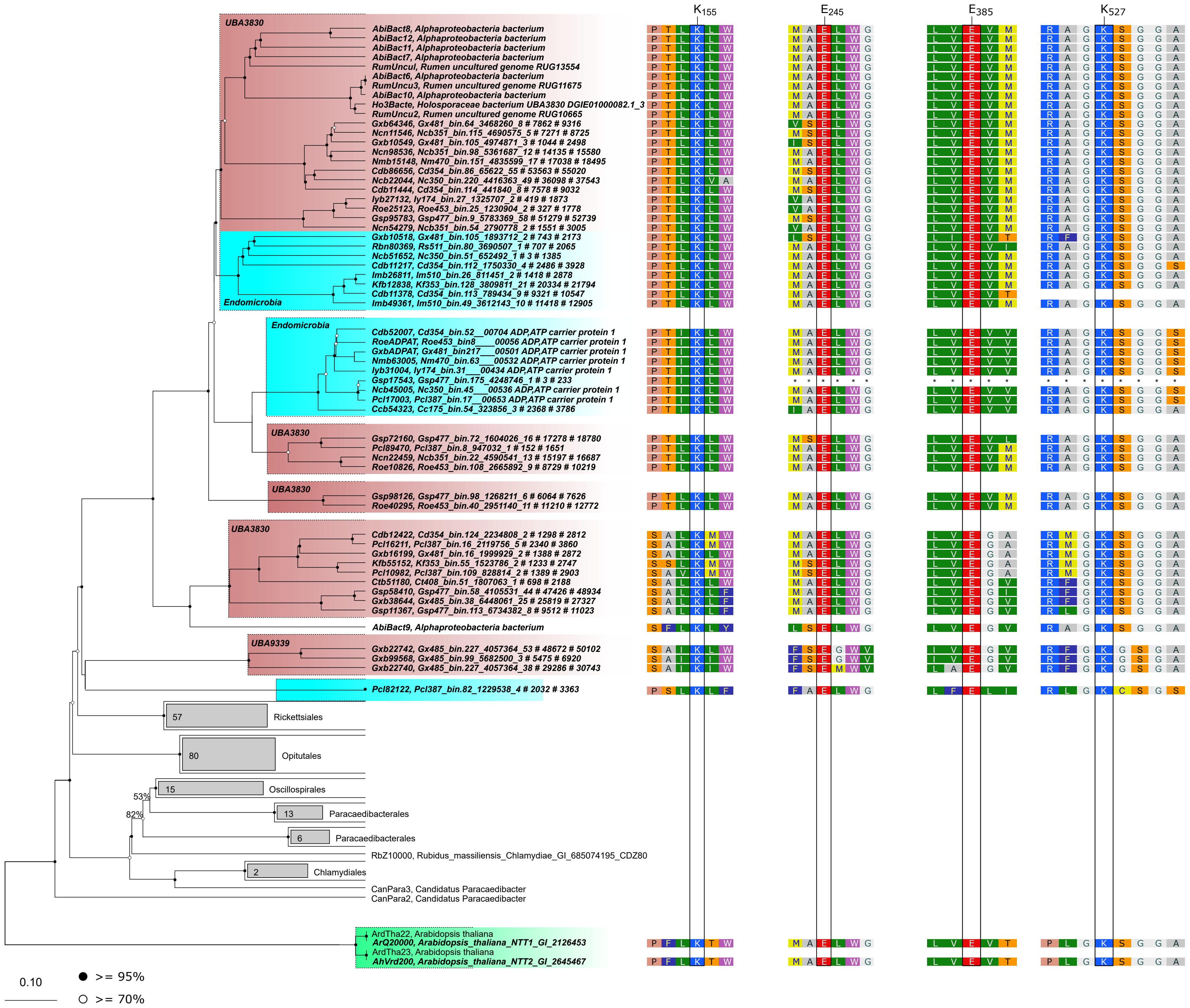

Figure S4: Phylogeny of the ADP/ATP antiporter of *Endomicrobiaceae* and its homologs in public databases. Bullets indicate node support (UFBoot; ●, ≥ 90; ○, ≥ 70%; 1,000 replicates). The scale bar indicates 0.1 amino acid substitutions per site. The four conserved residues that are critical for function and substrate specificity in the antiporter of *Arabidopsis thaliana* and their neighborhood in the alignment are shown.

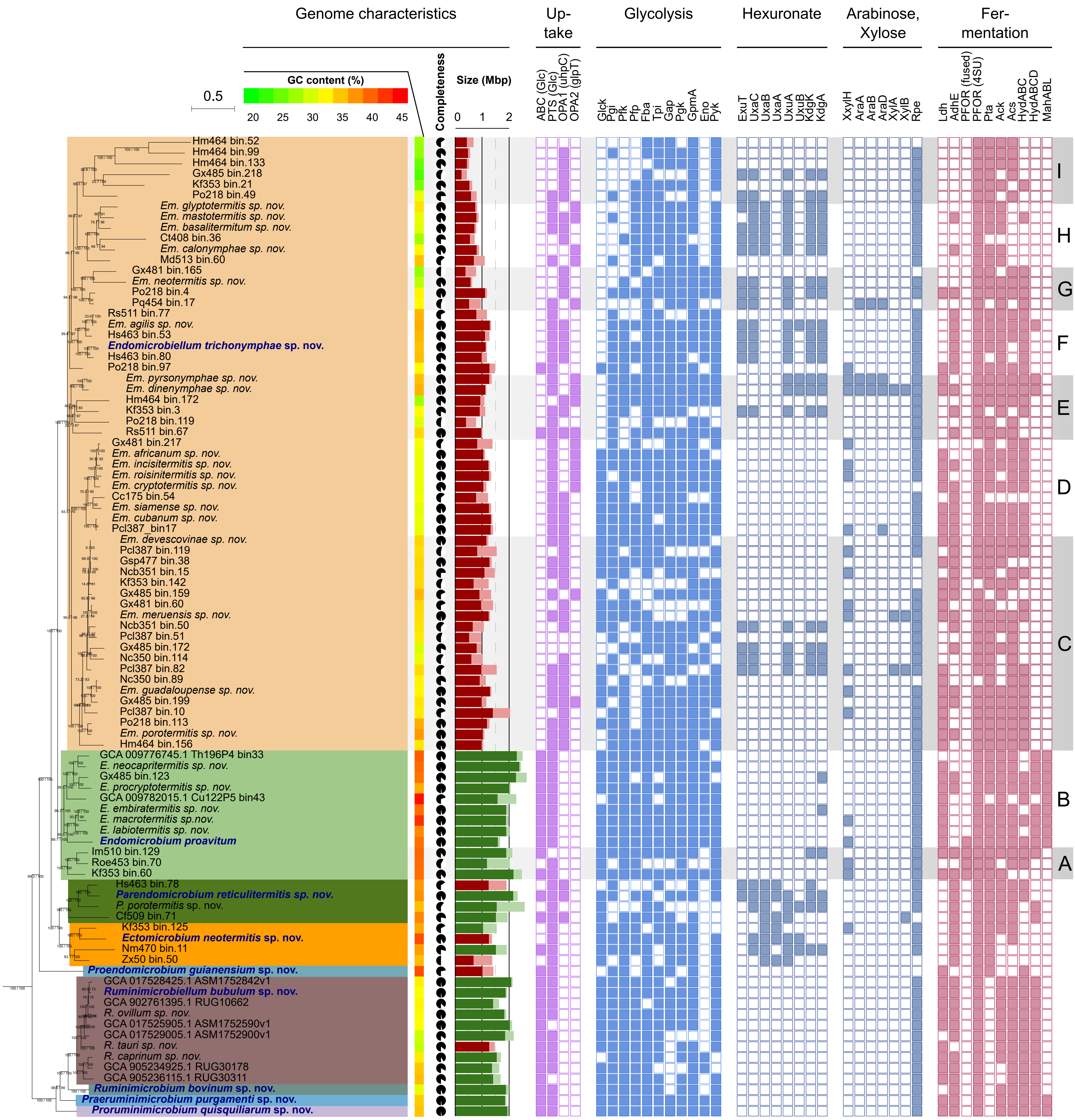

Figure S5: Expanded version of Figure 3, illustrating the key genes/pathways of catabolism encoded by the individual genomes of each lineage of *Endomicrobiaceae*. Non-standard abbreviations: ABC, ABC transporter; PTS, phosphotransferase system; OPA1, organophosphate:phosphate antiporter 1; OPA2, organophosphate:phosphate antiporter 2; Glck, glucokinase; Pgi, glucose-6-phosphate isomerase; Pfk, phosphofructokinase; Pfp, pyrophosphate–fructose 6-phosphate 1-phosphotransferase; Fba, fructose-bisphosphate aldolase; Tpi, triosephosphate isomerase; Gap, glyceraldehyde-3-phosphate dehydrogenase; Pgk, phosphoglycerate kinase; GpmA, phosphoglycerate mutase; Eno, enolase; Pyk, pyruvate kinase; ExuT, hexuronate transporter; UxaC, uronate isomerase; UxaB, altronate oxidoreductase; UxaA, altronate dehydratase; UxuA, mannonate dehydratase; UxuB, mannonate oxidoreductase; KdgK, 2-dehydro-3-deoxygluconokinase/2-dehydro-3-deoxygalactonokinase; KdgA, 2-dehydro-3-deoxy-phosphogluconate/2-dehydro-3-deoxy-6-phosphogalactonate aldolase; XylH, xylose/arabinose transport system; AraA, arabinose isomerase; AraB, ribulokinase; XylA, xylose isomerase; XylB, xylulose kinase; Rpe, ribulose-phosphate 3-epimerase; Ldh, lactate dehydrogenase; AdhE, bifunctional aldehyde–alcohol dehydrogenase; PFOR, pyruvate:ferredoxin/flavodoxin oxidoreductase; Pta, phosphate acetyltransferase; Ack, acetate kinase; HydABC, trimeric [FeFe] hydrogenase; HydABCD, tetrameric [FeFe] hydrogenase; MahABL; [NiFe] hydrogenase.

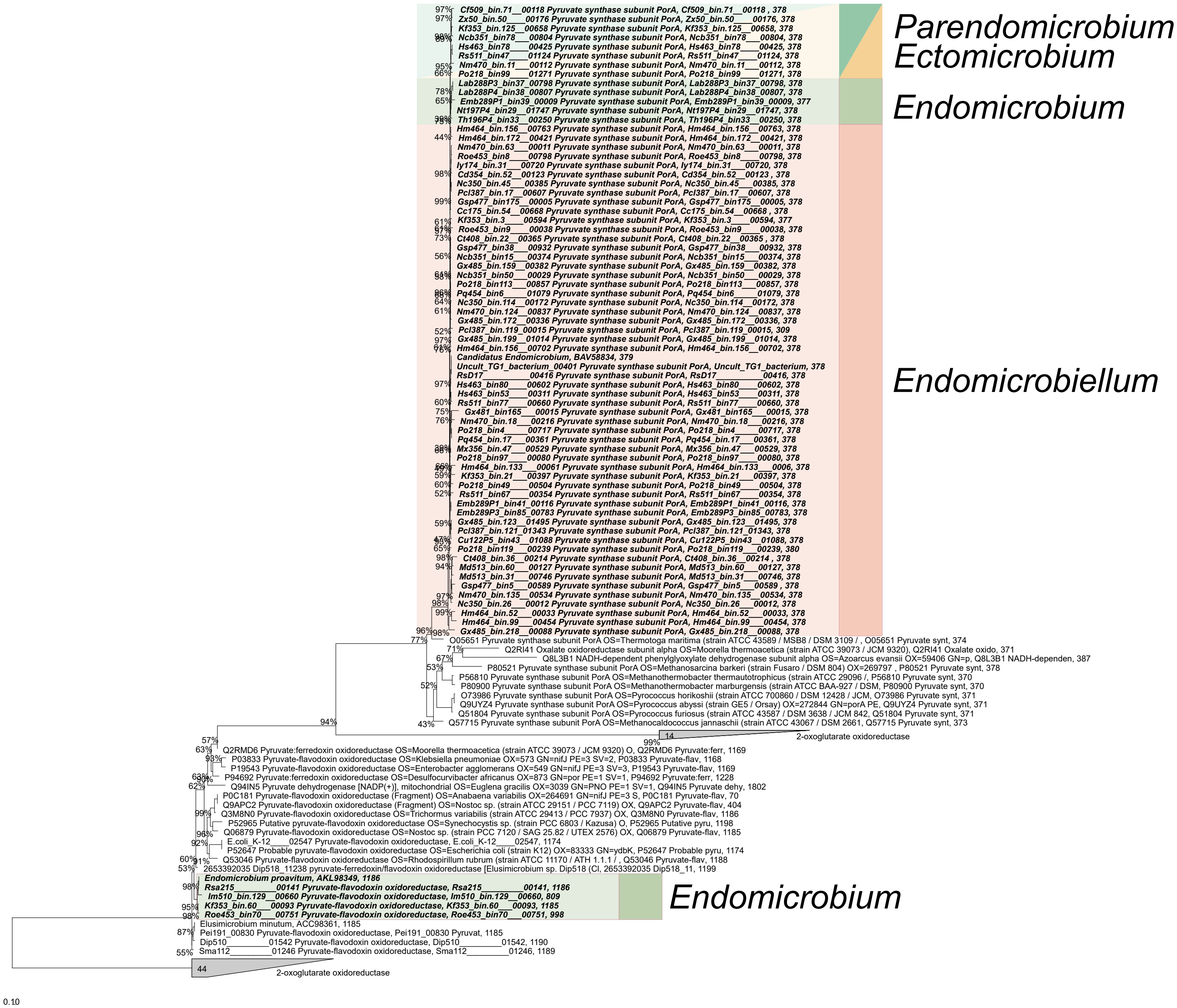

Figure S6: Phylogeny of the pyruvate:ferredoxin oxidoreductase of *Endomicrobiaceae* and its homologs in public databases**.** The consensus tree was inferred from a LG+R5 model of evolution and based on a curated alignment of 323 amino acid positions and is rooted with the beta subunit of the 2-oxoglutarate oxidoreductase (korB). Bullets indicate node support (UFBoot; ●, ≥ 90; ○, ≥ 70%; 1,000 replicates). The scale bar indicates 0.1 amino acid substitutions per site.

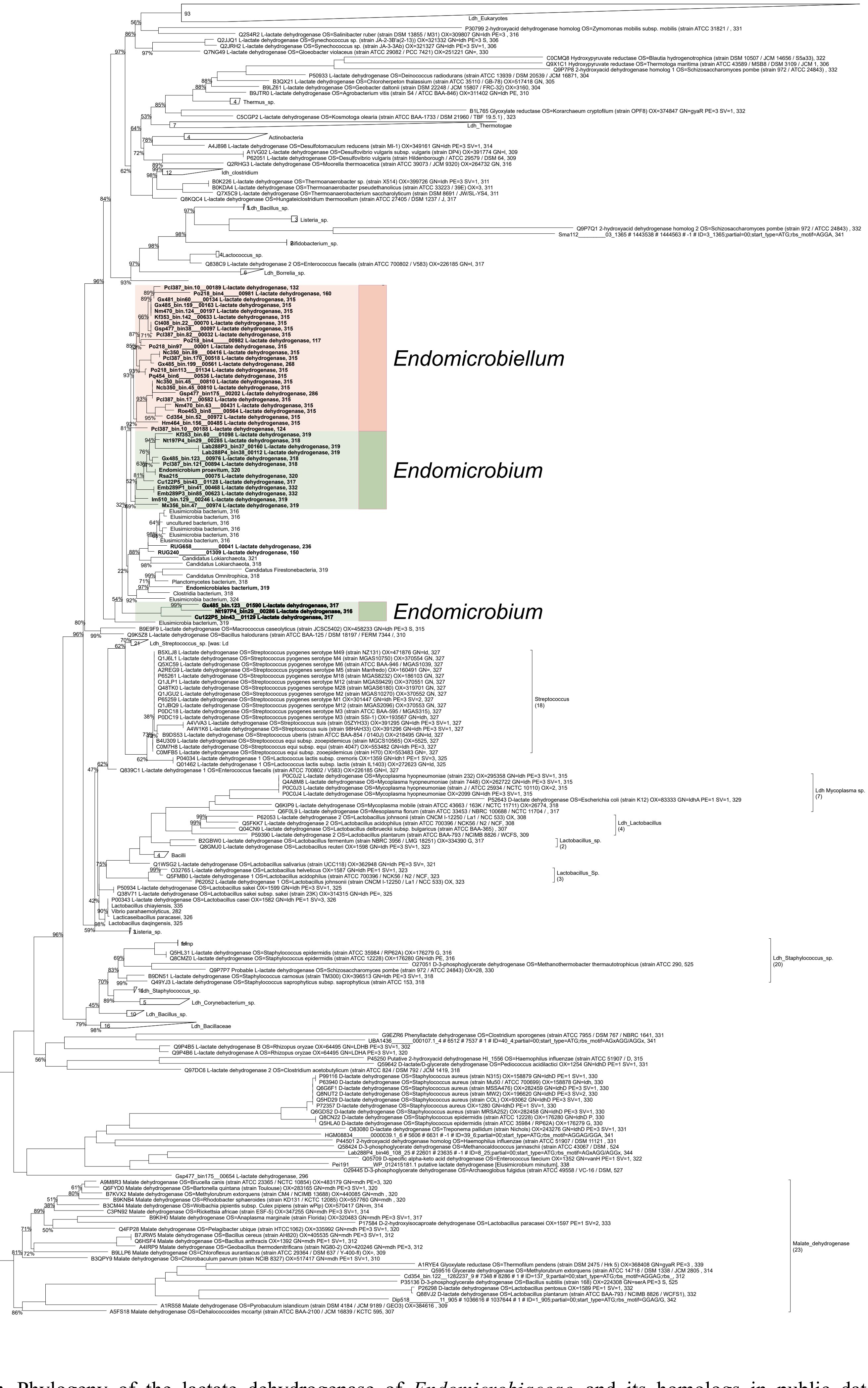

Figure S7: Phylogeny of the lactate dehydrogenase of *Endomicrobiaceae* and its homologs in public databases. The consensus tree is inferred from on a LG+G4 model of evolution and is based on 140 amino acid positions. The tree was rooted with malate dehydrogenase sequences. Bullets indicate node support (UFBoot; ●, ≥ 90; ○, ≥ 70%; 1,000 replicates). The scale bar indicates 0.1 nucleotide substitutions per site.

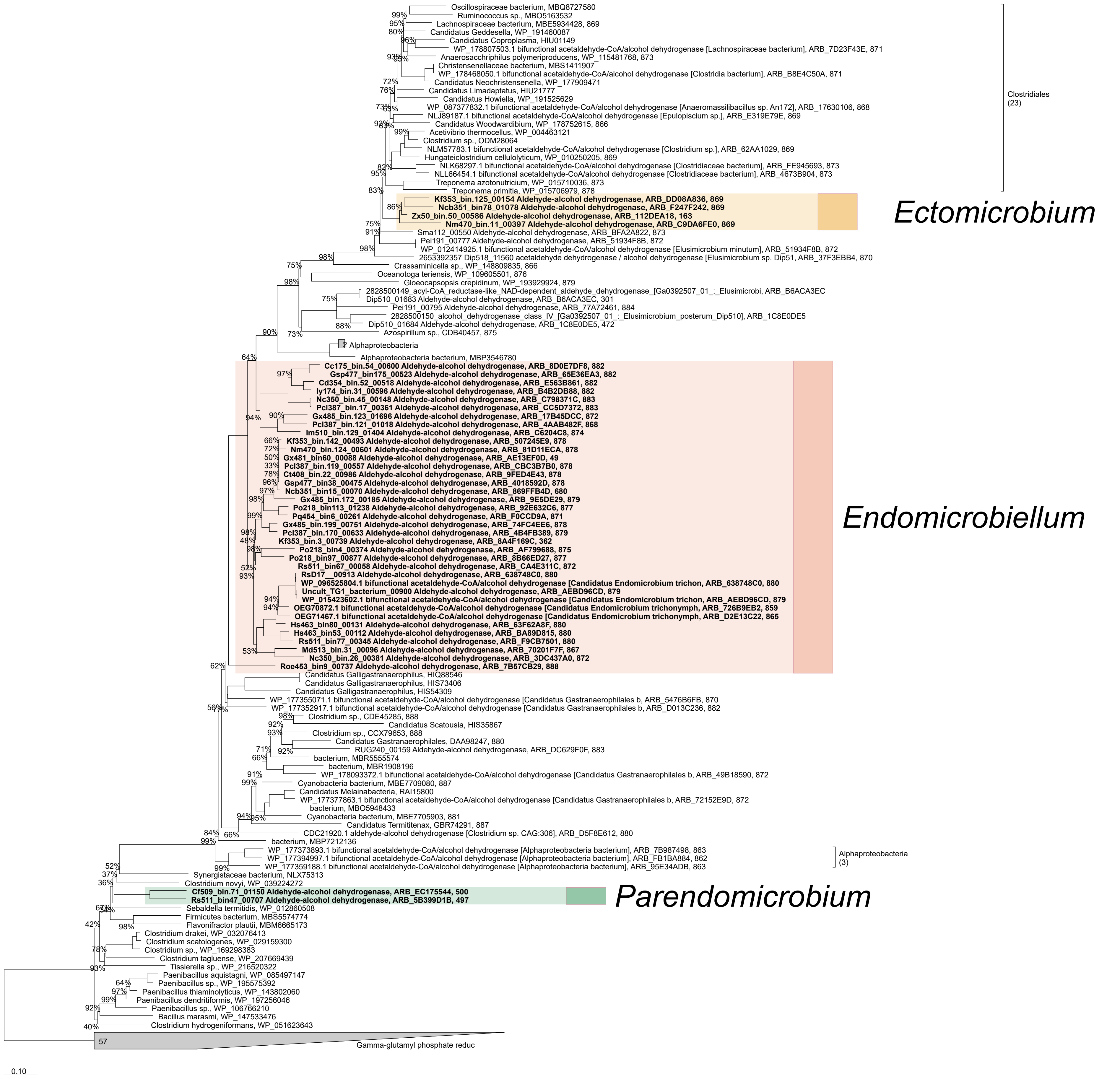

Figure S8: Phylogeny of the bifunctional aldehyde/ethanol dehydrogenase of *Endomicrobiaceae* and its homologs in public databases. The tree was inferred from a LG+F+R5 model of evolution and is based on alignment of 608 unambiguously aligned amino acid positions. It is rooted with gamma-glutamyl phosphate reductase sequences. Bullets indicate node support (UFBoot; ≥ ,●90○ ;, ≥ 70%; 1,000 replicates). The scale bar indicates 0.1 amino acid substitutions per site.

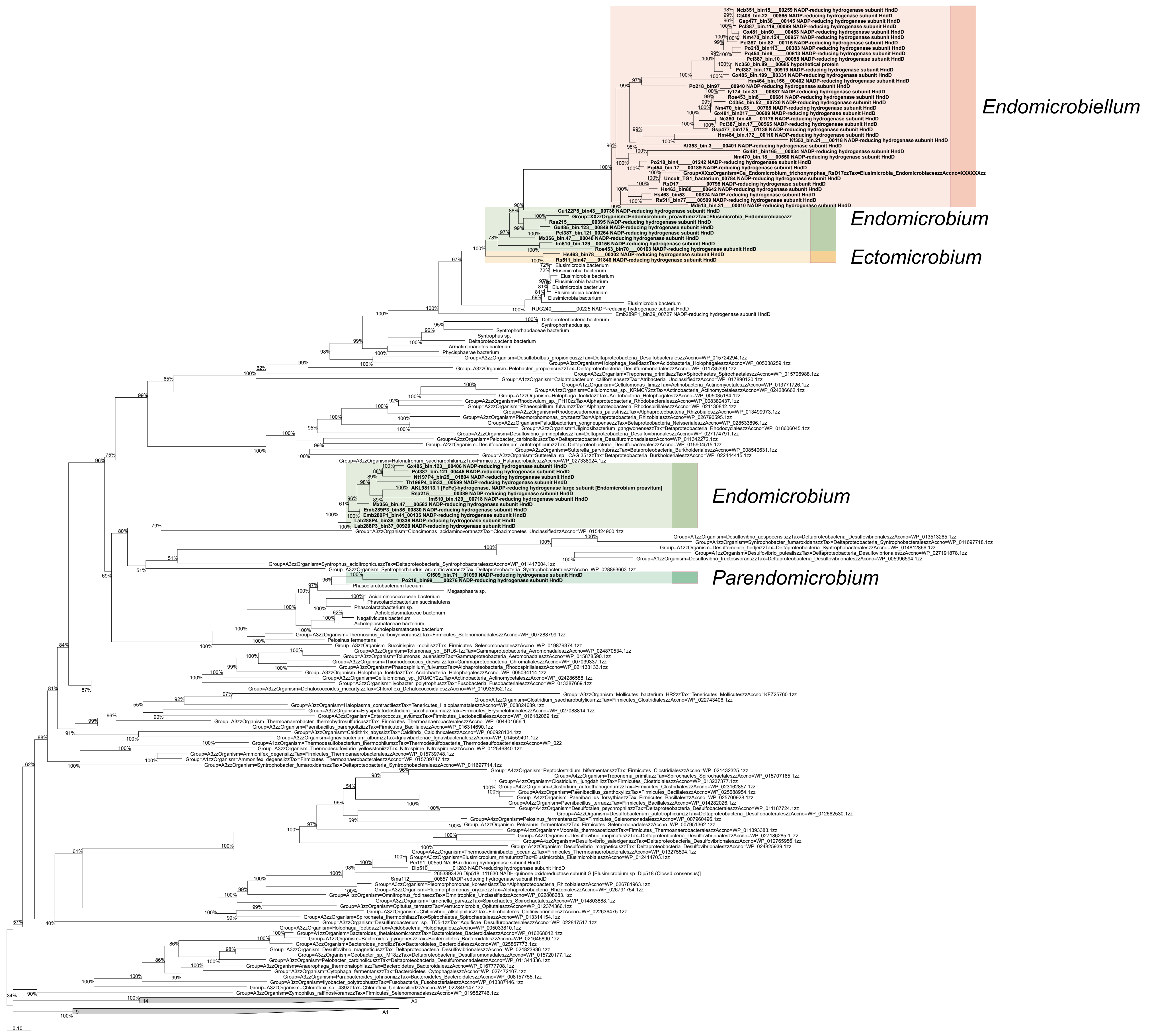

Figure S9: Phylogeny of the [FeFe]-hydrogenases of *Endomicrobiaceae* and their homologs in public databases. The consensus tree is based on an LG+R7 model of evolution of a curated alignment of 281 amino acid positions. Bullets indicate node support (UFBoot; ●, ≥ 90; ○, ≥ 70%; 1,000 replicates). The scale bar indicates 0.1 amino acid substitutions per site. The tree is rooted with hydrogenase group A4.

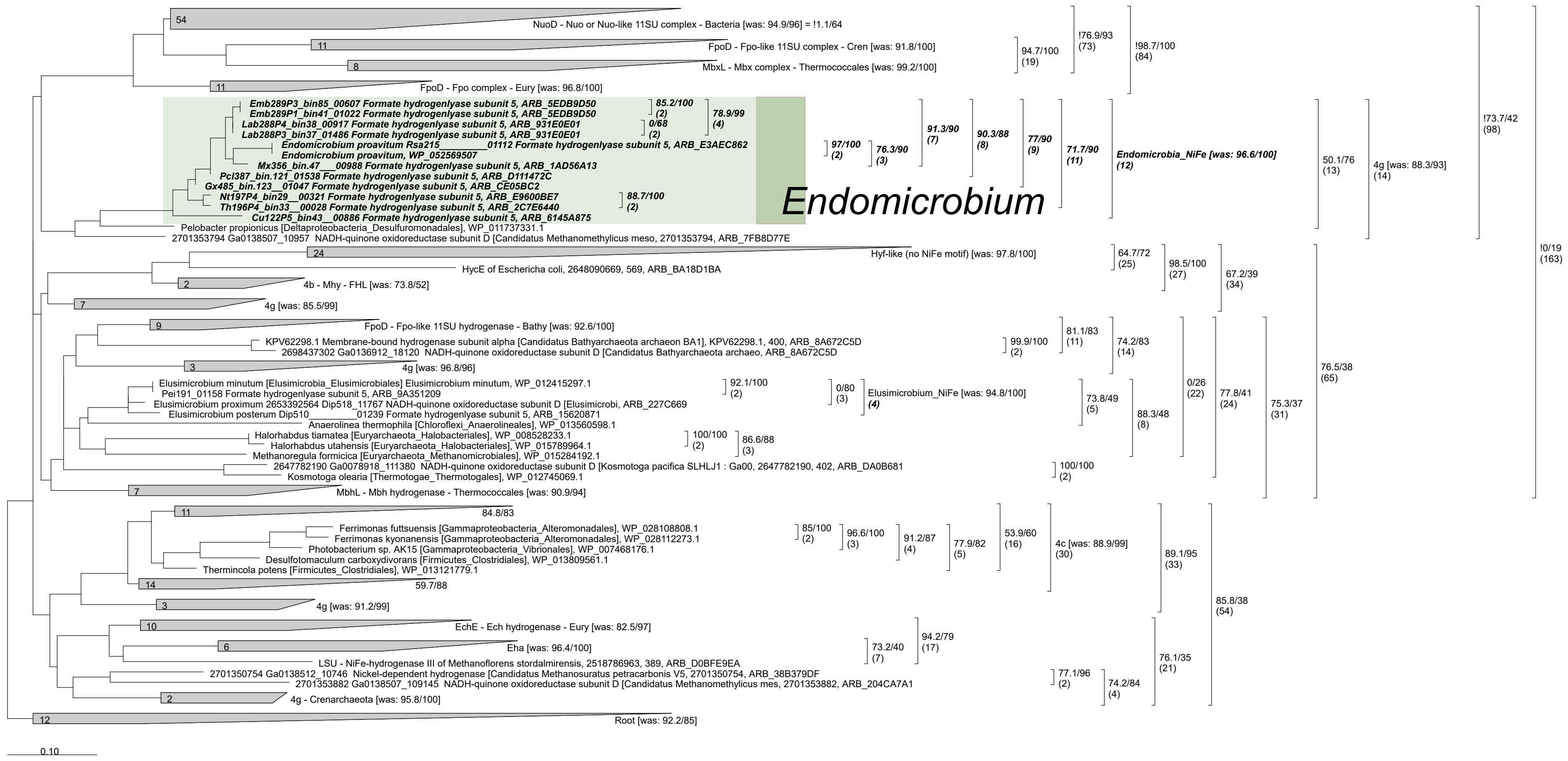

Figure S10: Phylogeny of the catalytic subunit of the [NiFe]-hydrogenase of *Endomicrobiaceae* and its homologs in public databases. The phylogenetic tree was inferred from a LG+R6 model of evolution based on a curated alignment of 133 amino acid positions. Bullets indicate node support (UFBoot; ●, ≥ 90; ○, ≥ 70%; 1,000 replicates). The tree is rooted with the archaeal subgroups 4h and 4i. The scale bar indicates 0.1 amino acid substitutions per site.

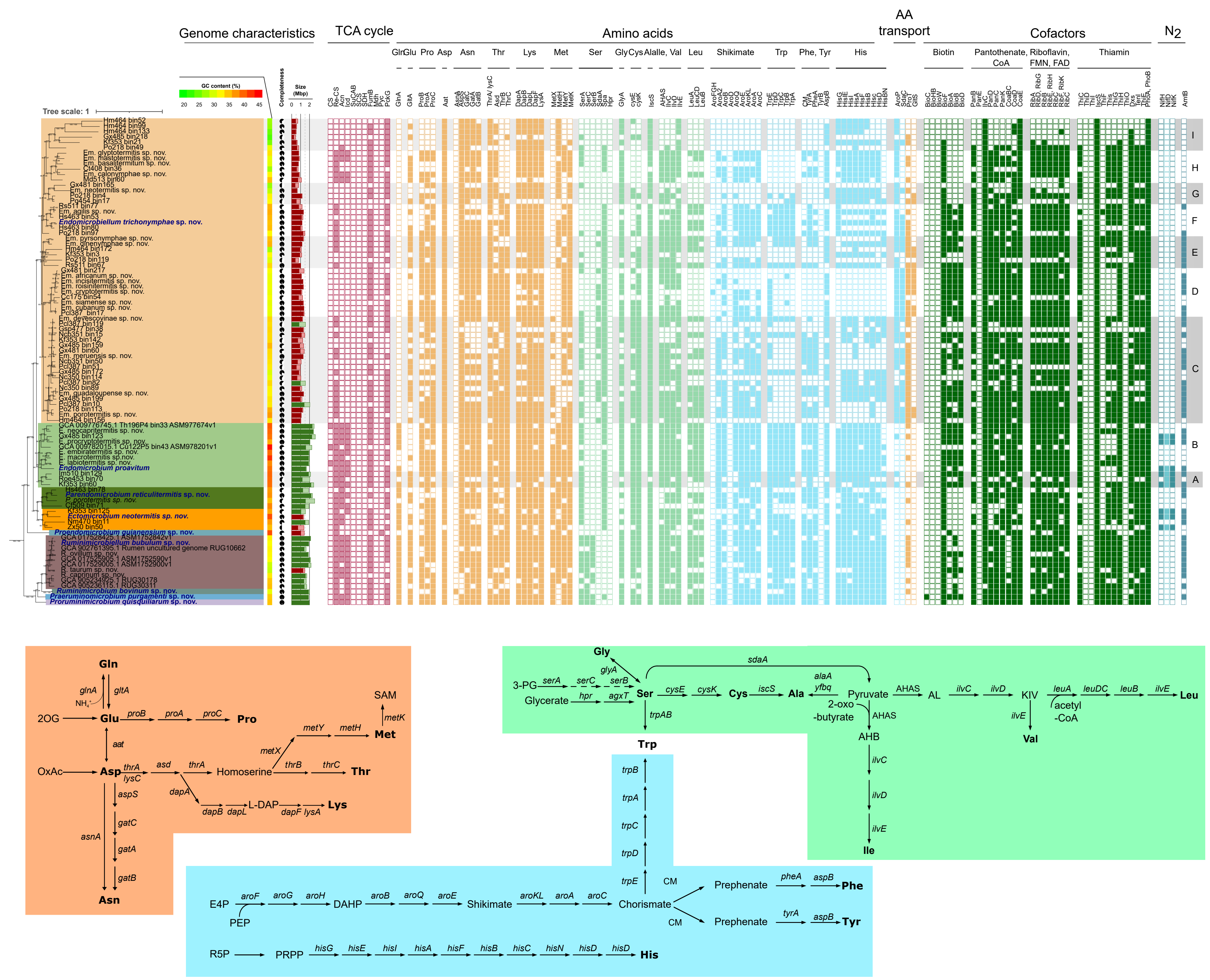

Figure S11: Expanded version of Figure 3, illustrating the key genes/pathways of important anabolic pathways encoded by the individual genomes of each lineage of *Endomicrobiaceae*. The biosynthetic pathways for amino acids that derive from the TCA cycle (blue background), the pentose phosphate pathway (orange background), and glycolysis (light green background) can be found at the bottom of the figure. Non-standard abbreviations: CS, citrate synthase; , Re-CS, *Re*-citrate synthase; Acn, aconitase; Icd, isocitrate dehydrogenase; SucAB, 2-oxoglutarate dehydrogenase; SCS, succinyl-coA synthetase; SDH, succinate dehydrogenase; FumB, fumarase; Mdh, malate dehydrogenase; Pyc, pyruvate carboxylase; PckG, PEP carboxykinase; GlnA, glutamine synthetase; GltA, glutamate synthase; ProB, glutamate 5-kinase; ProA, gamma-glutamyl phosphate reductase; ProC, pyrroline-5-carboxylate reductase; Aat, aspartate aminotransferase; AsnA, aspartate–ammonia ligase; AspS, aspartate–tRNA(Asp/Asn) ligase; GatC, aspartyl/glutamyl-tRNA(Asn/Gln) amidotransferase subunit C; GatA, aspartyl/glutamyl-tRNA(Asn/Gln) amidotransferase subunit A; GatB, aspartyl/glutamyl-tRNA(Asn/Gln) amidotransferase subunit B; ThrA, bifunctional aspartokinase/homoserine dehydrogenase; AsD, bifunctional aspartate aminotransferase/L-aspartate beta-decarboxylase; ThrB, homoserine kinase; ThrC, threonine synthase; DapA, 4-hydroxy-tetrahydrodipicolinate synthase; DapB, 4-hydroxy-tetrahydrodipicolinate reductase; DapL, LL-diaminopimelate aminotransferase; DapF, diaminopimelate epimerase; LysA, diaminopimelate decarboxylase; MetX, homoserine O-acetyltransferase; MetH, methionine synthase; MetY, O-acetyl-L-homoserine sulfhydrylase; MetK, S-adenosylmethionine synthase; SerA, D-3-phosphoglycerate dehydrogenase; SerC, phosphoserine aminotransferase; SerB, phosphoserine phosphatase; Spa, serine:pyruvate aminotransferase; Hpr, hydroxypyruvate reductase; GlyA, serine hydroxymethyltransferase; CysE, serine acetyltransferase; CysK, cysteine synthase A; IscS, cysteine desulfurase; AHAS, acetolactate synthase; IlvC, ketol-acid reductoisomerase; IlvD, dihydroxy-acid dehydratase; IlvE, branched-chain-amino-acid transaminase; LeuA, 2-isopropylmalate synthase; LeuCD, 3-isopropylmalate dehydratase; LeuB, 3-isopropylmalate dehydrogenase; AroFGH, phospho-2-dehydro-3-deoxyheptonate aldolase; AroA, 3-phosphoshikimate 1-carboxyvinyltransferase; AroB, 3-dehydroquinate synthase; AroD, 3-dehydroquinate dehydratase, type I; AroQ, 3-dehydroquinate dehydratase, type II; AroE, shikimate dehydrogenase; AroKL; AroC, chorismate synthase; TrpE, anthranilate synthase; TrpD, anthranilate phosphoribosyltransferase; TrpC, indole-3-glycerol phosphate synthase; TrpB, tryptophan synthase, beta subunit; TrpA, tryptophan synthase, alpha subunit; CM, chorismate mutase; TyrA, prephenate dehydrogenase; PheA, prephenate dehydratase; TyrB, Tyrosine aminotransferase; AspB, aspartate aminotransferase; HisG, ATP phosphoribosyltransferase; HisIE, bifunctional phosphoribosyl-ATP diphosphatase/ cyclohydrolase; HisI, Phosphoribosyl-AMP cyclohydrolase; HisA, 1-(5-phosphoribosyl)-5-[(5-phosphoribosylamino)methylideneamino] imidazole-4-carboxamide isomerase; HisF, imidazole glycerol phosphate synthase catalytic subunit; HisB, imidazoleglycerol-phosphate dehydratase; HisC, histidinol-phosphate aminotransferase; HisD, histidinol dehydrogenase; HisBN, bifunctional histidinol-phosphatase/imidazoleglycerol-phosphate dehydratase; AroP, aromatic amino acid transport protein; SdaC, serine transporter; ProT, proline transporter; GltS, sodium/glutamate symporter; BioC, Malonyl-[acyl-carrier protein] O-methyltransferase; BioHB, pimelyl-[acyl-carrier protein] methyl ester esterase; BioW, 6-carboxyhexanoate–CoA ligase; BioF, 8-amino-7-oxononanoate synthase; BioA, adenosylmethionine-8-amino-7-oxononanoate transaminase; BioB, biotin synthase; BioD, ATP-dependent dethiobiotin synthetase; PanB, 3-methyl-2-oxobutanoate hydroxymethyltransferase; abpB, 2-dehydropantoate 2-reductase; PanD, L-aspartate-alpha-decarboxylase; PanC, pantoate–beta-alanine ligase; PanK, pantothenate kinase; CoaBC, bifunctional phosphopantothenoylcysteine synthetase/decarboxylase; CoaD, pantetheine-phosphate adenylyltransferase; CoaE, dephospho-CoA kinase; RibA, GTP cyclohydrolase II; RibDG, riboflavin-specific deaminase; RibB, 3,4-dihydroxy-2-butanone 4-phosphate synthase; RibEH, 6,7-dimethyl-8-ribityllumazine synthase; RibC, riboflavin synthase; RibFK, bifunctional riboflavin kinase/FMN adenylyltransferase; ThiC, phosphomethylpyrimidine synthase; ThiD, hydroxymethylpyrimidine/phosphomethylpyrimidine kinase; ThiI, tRNA sulfurtransferase; ThiF, thiazole biosynthesis adenylyltransferase; ThiS, sulfur carrier protein; ThiG, thiazole biosynthesis protein; ThiH, 2-iminoacetate synthase; ThiO, glycine oxidase; Dxs, 1-deoxy-D-xylulose-5-phosphate synthase; TenI, thiamine monophosphate synthase; ThiE, thiamine-phosphate diphosphorylase; PhoAB, alkaline phosphatase; NifH, nitrogenase iron protein; NifD, nitrogenase molybdenum-iron protein alpha chain; NifK, nitrogenase molybdenum-iron protein beta chain; AmtB, ammonium transporter.

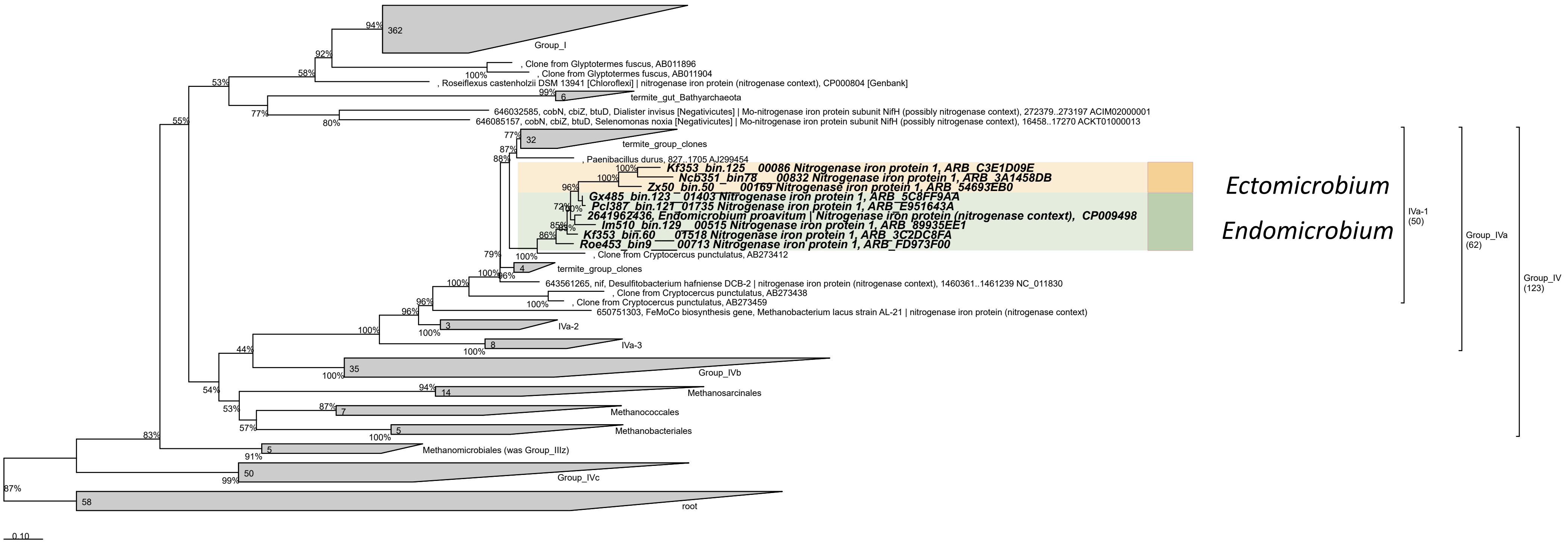

Figure S12: Phylogeny of nitrogenase reductase (NifH) of *Endomicrobiaceae* and its homologs in public databases. The tree was inferred under an LG+R10 model of evolution and is based on 275 unambiguously aligned amino acid positions. Other sequences (with only arb accession numbers) were obtained in a previous study of our lab and are not yet published. The tree is rooted with the protochlorophyllide reductase (Bcl and Chil). Bullets indicate node support (UFBoot; ●, ≥ 90; ○, ≥ 70%; 1,000 replicates).

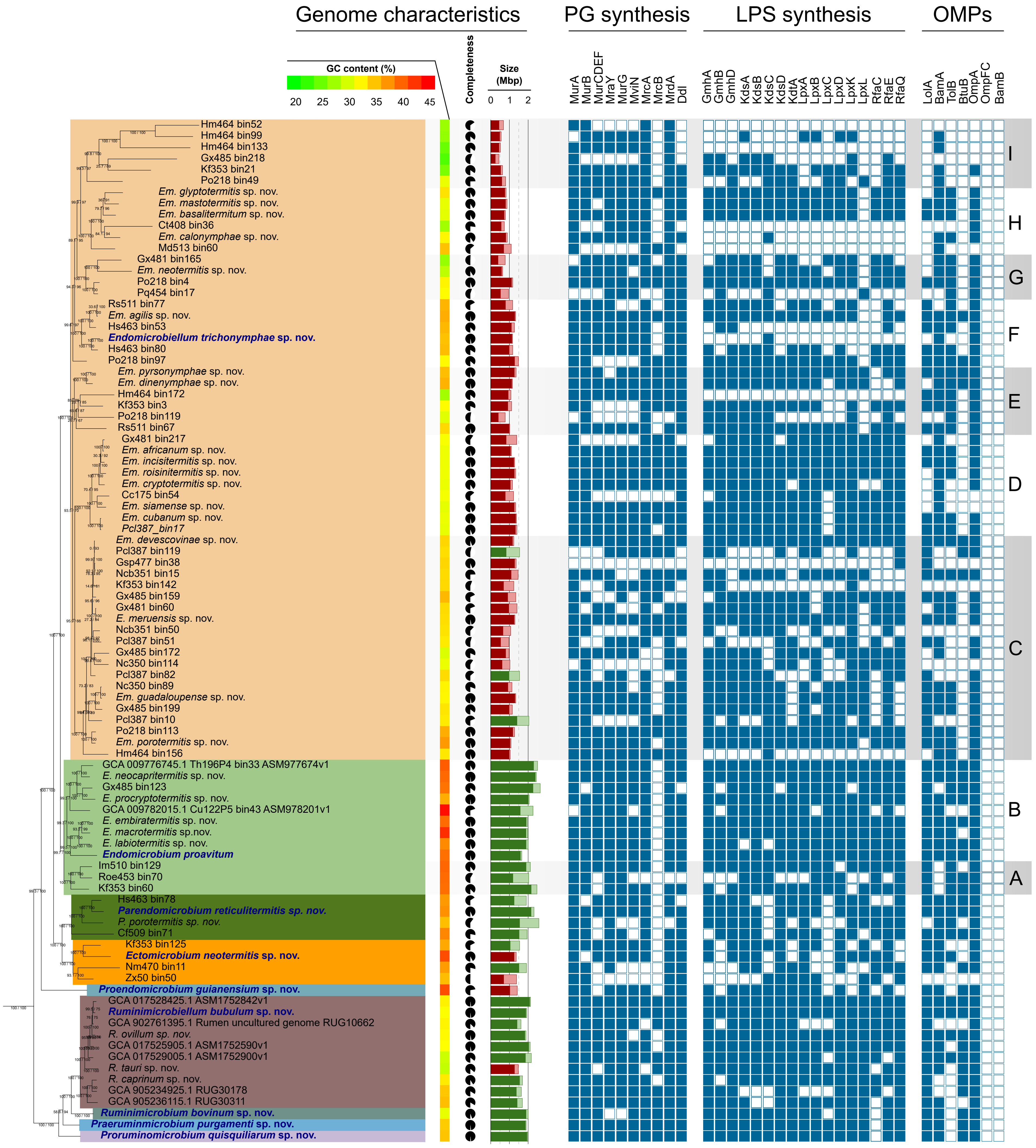

Figure S13: Extended version of Figure 3, illustrating key genes/pathways for components of the cell envelope (PG, peptidoglycan; LPS, lipopolysaccharide; OMPs, outer membrane proteins) encoded by the individual genomes of each lineage of *Endomicrobiaceae*. key genes/pathways encoded in the individual genomes of each lineage. Non-standard abbreviations: MurA, UDP-N-acetylglucosamine 1-carboxyvinyltransferase; MurB, UDP-N-acetylenolpyruvoylglucosamine reductase; MurCDEF, UDP-N-acetylmuramoylalanine–D-glutamate ligase; MraY, phospho-N-acetylmuramoyl-pentapeptide-transferase; MurG, UDP-N-acetylglucosamine–N-acetylmuramyl-(pentapeptide) pyrophosphoryl-undecaprenol N-acetylglucosamine transferase; MviN, probable peptidoglycan biosynthesis protein; MrcA, penicillin-binding protein 1A; MrcB, penicillin-binding protein 1B; MrdA, peptidoglycan D, D-transpeptidase; Ddl, D-alanine–D-alanine ligase; GmhA, phosphoheptose isomerase; GmhB, D-glycero-beta-D-manno-heptose-1,7-bisphosphate 7-phosphatase; GmhD, GDP-D-glycero-alpha-D-manno-heptose dehydrogenase; KdsA, 2-dehydro-3-deoxyphosphooctonate aldolase; KdsB, 3-deoxy-manno-octulosonate cytidylyltransferase; KdsC, 3-deoxy-D-manno-octulosonate 8-phosphate phosphatase; KdsD, arabinose 5-phosphate isomerase; KdtA, 3-deoxy-D-manno-octulosonic acid transferase; LpxA, acyl-[acyl-carrier-protein]–UDP-N-acetylglucosamine O-acyltransferase; LpxB, lipid-A-disaccharide synthase; LpxC, UDP-3-O-acyl-N-acetylglucosamine deacetylase; LpxK, tetraacyldisaccharide 4'-kinase, LpxL, lipid A biosynthesis lauroyltransferase; RfaC, lipopolysaccharide heptosyltransferase 1; RfaE, bifunctional protein HldE; RfaQ, lipopolysaccharide core heptosyltransferase; LolA, outer-membrane lipoprotein carrier protein; BamA, outer membrane protein assembly factor; TolB, Tol-Pal system protein; BtuB, vitamin B12 transporter; OmpA, outer membrane protein A; OmpFC, outer membrane porin; BamB, outer membrane protein assembly factor. Outer membrane proteins absent in the entire family *Endomicrobiaceae* were omitted for clarity.

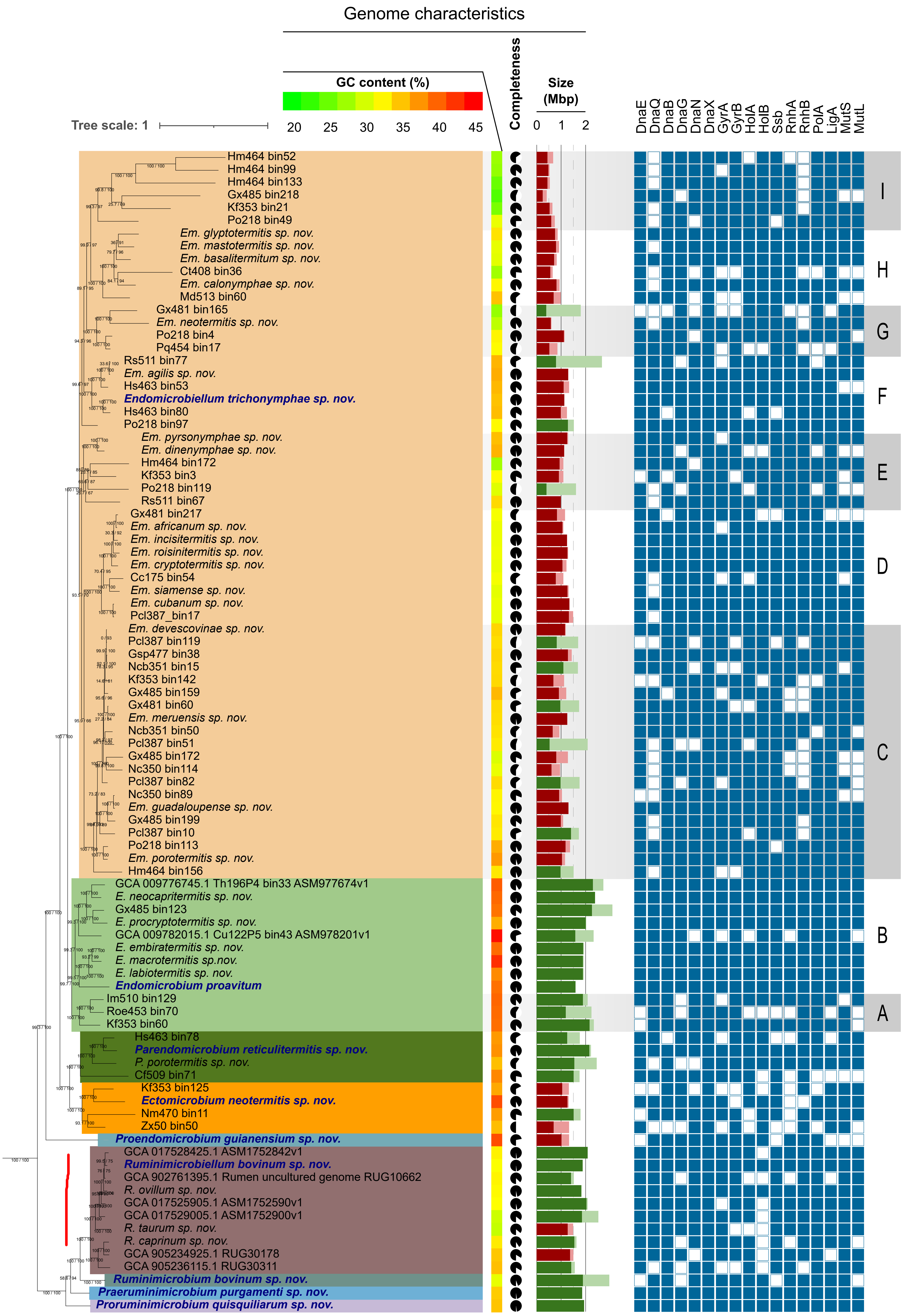

Figure S14: Extended version of Figure 3, illustrating the key genes involved in informational processing encoded by the individual genomes of each lineage of *Endomicrobiaceae*. Non-standard abbreviations: DnaE, DNA polymerase III subunit alpha; DnaQ, DNA polymerase III subunit epsilon; DnaB, replicative DNA helicase; DnaG, DNA primase; DnaN, beta sliding clamp; DnaX, DNA polymerase III subunit tau; GyrA, DNA gyrase subunit A; GyrB, DNA gyrase subunit B; HolA, DNA polymerase III subunit delta; HolB, DNA polymerase III subunit delta'; Ssb, single-strand DNA-binding protein; RnhA, ribonuclease HI; RnhB, ribonuclease HII; PolA, DNA polymerase I; LigA, DNA ligase; MutS, DNA mismatch repair protein MutS; MutL, DNA mismatch repair protein MutL.

Figure S15 (HTML File, open link in browser)

Figure S15: Interactive map showing genome size and GC content of the MAGs from termite guts, comparing lineages that comprise established and putative endosymbionts (in color) with their closest relatives (in gray). The origin of the MAGs from lower termites (●) and higher termites (○) is indicated; the genomes of well-characterized endosymbionts are marked. The identity of the MAG is revealed by hovering the cursor over the bullets.

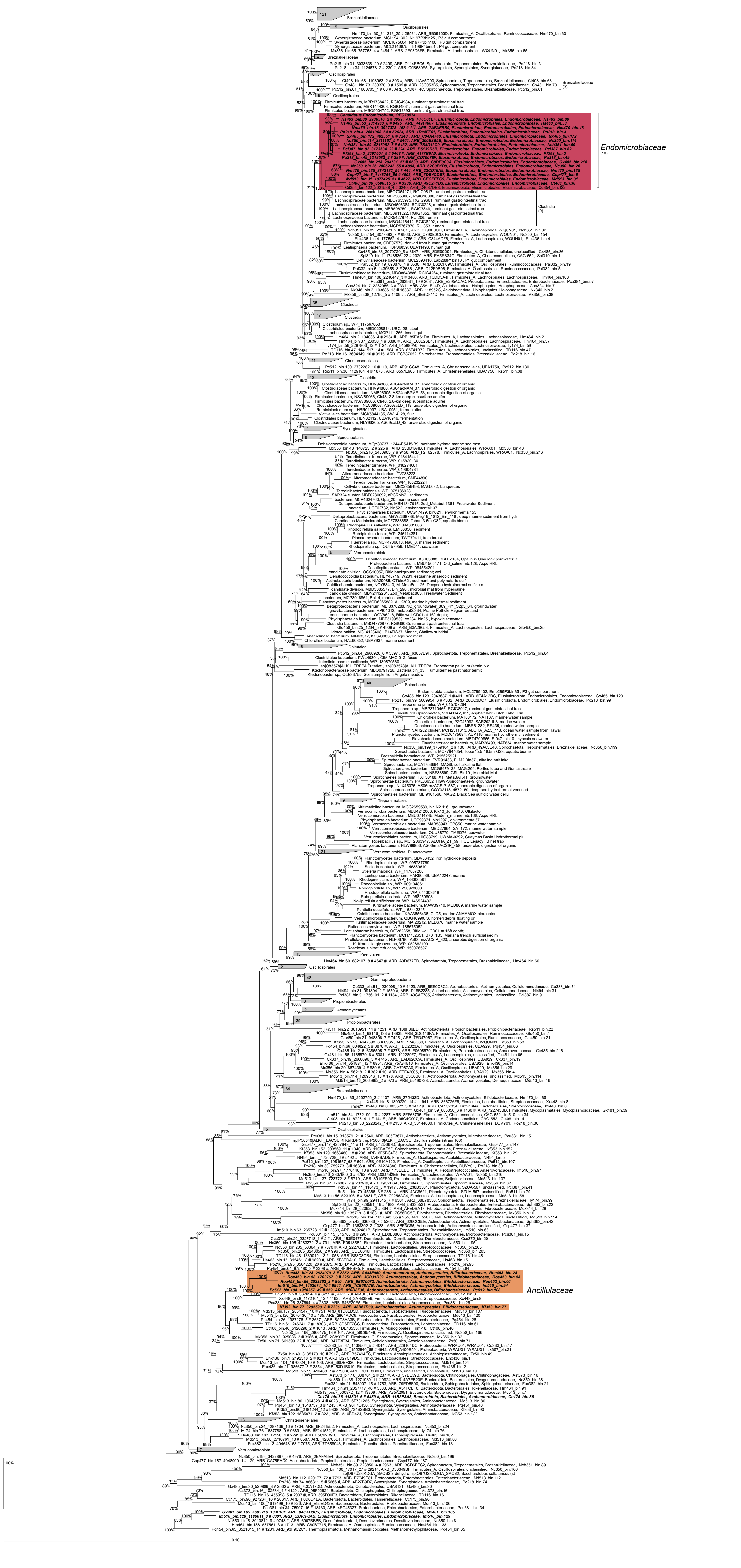

Figure S16: Phylogeny of the KDPG aldolase (KdgA) of *Endomicrobiaceae* and its homologs in public databases. The tree was inferred under an LG+R10 model of evolution and is based on 1,298 unambiguously aligned amino acid positions. Bullets indicate node support (UFBoot; ●, ≥ 90; ○, ≥ 70%; 1,000 replicates). The scale bar indicates 0.1 amino acid substitutions.

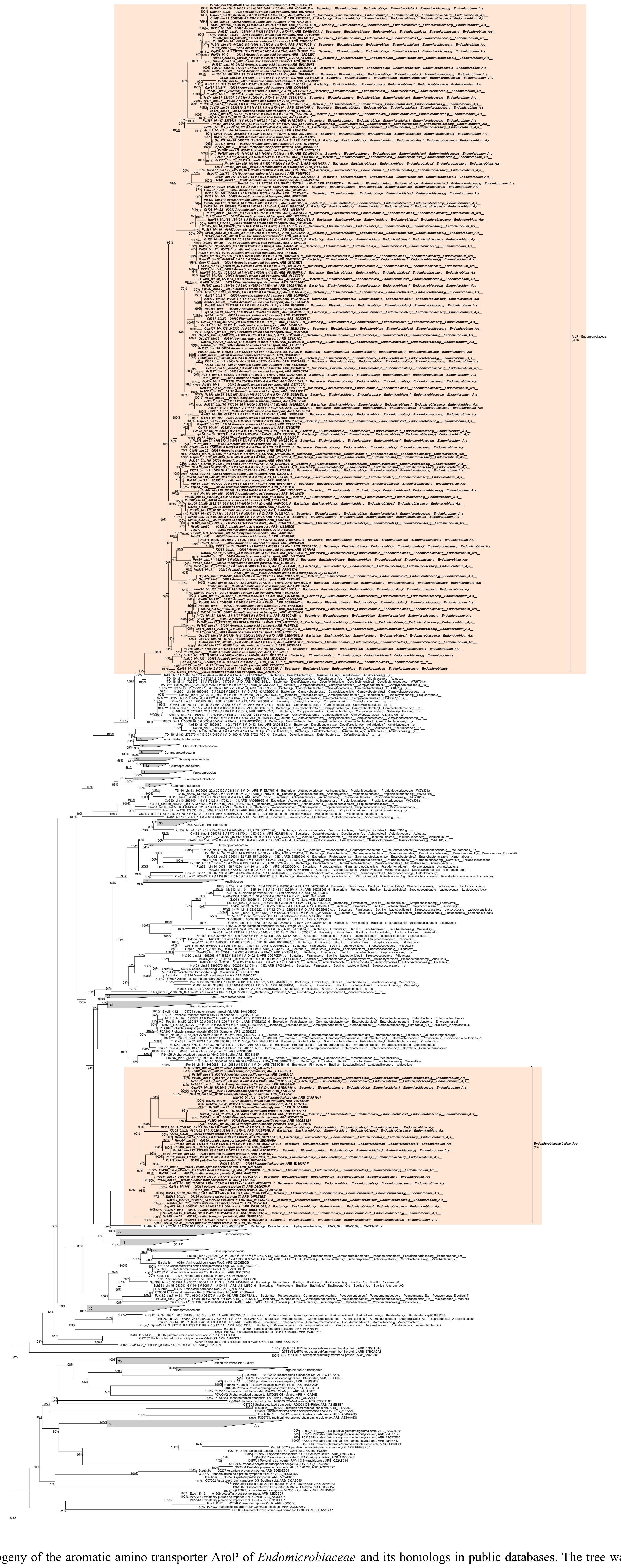

Figure S17: Phylogeny of the aromatic amino transporter AroP of *Endomicrobiaceae* and its homologs in public databases. The tree was inferred under an LG+F+R10 model of evolution and is based on 1,242 unambiguously aligned amino acid positions. Bullets indicate node support (UFBoot; ●, ≥ 90; ○, ≥ 70%; 1,000 replicates). The scale bar indicates 0.1 amino acid substitutions.
